# Circulating Extracellular Vesicle Protein Biomarkers for the Early Detection of High-Grade Serous Ovarian Cancer

**DOI:** 10.1016/j.mcpro.2026.101508

**Published:** 2026-01-08

**Authors:** Sagar Rayamajhi, Jared Sipes, Bidii Ngala, Amrita Mitra, Meizhang Li, Camille V. Trinidad, Wei Cui, Mohammod Mahmudur Rahman, Foyez Ahmmed, Leonidas E. Bantis, Mihaela E. Sardiu, Dennis W. Province, Harsh B. Pathak, Andrew K. Godwin

**Affiliations:** 1 Department of Pathology and Laboratory Medicine, University of Kansas Medical Center, Kansas City, Kansas, USA; 2 Bioengineering Program, The University of Kansas, Lawrence, Kansas, USA; 3 Department of Biomedical Engineering, Vanderbilt University, Nashville, Tennessee, USA; 4 Department of Biostatistics and Data Science, University of Kansas Medical Center, Kansas City, Kansas, USA; 5 Department of Biochemistry and Microbiology, University of Arkansas for Medical Science, Little Rock, Arkansas, USA; 6 Kansas Institute for Precision Medicine, University of Kansas Medical Center, Kansas City, Kansas, USA; 7 University of Kansas Cancer Center, University of Kansas Medical Center, Kansas City, Kansas, USA

**Keywords:** diagnostic biomarker, early cancer detection, extracellular vesicles, high-grade serous ovarian cancer, liquid biopsy biomarker, proteomics

## Abstract

Small extracellular vesicles (sEVs), lipid-bilayer delimited particles (50–200 nm) released by cells, are emerging as a promising class of liquid biopsy biomarkers for elusive cancers, such as high-grade serous ovarian cancer (HGSOC). HGSOC originates from the fallopian tube (FT), progressing from p53 signatures to a precursor lesion known as serous tubal intraepithelial carcinoma (STIC). We hypothesize that sEVs contribute to ovarian cancer pathogenesis, carry cargo reflective of their site of origin, and serve as diagnostic biomarkers for early detection. To test this, we established a case–control cohort using archival plasma samples from 30 HGSOC patients (10 early stage [ES] and 20 late stage [LS]) and 40 healthy controls (HC). sEVs were enriched by size-exclusion chromatography and profiled by LC–MS/MS. Across all samples, 1078 EV-associated proteins (exoproteins) were identified, including 52 upregulated in ES HGSOC *versus* HC and 59 upregulated in LS HGSOC *versus* HCs (log_2_ fold change >1, *p* < 0.05). Upregulated EV proteins were prioritized based on FT origin and tissue expression in STIC lesions. Seven candidate biomarkers (MYL6, GSTP1, TTYH3, PRDX6, MUC1, MYH14, and PTGS1) were validated by immunohistochemistry in FT tissue harboring STIC lesions and in HGSOC tissues, as well as by Western blotting in FT/HGSOC cell–derived EVs. These findings suggest that circulating exoproteins upregulated in ES cancer disease reflect precursor lesions. A four-protein combinatorial panel (MUC1, MYL6, TTYH3, and GSTP1), selected using Akaike Information Criterion, yielded an area under the curve (AUC) of 0.975 and 90% sensitivity at 95% specificity for distinguishing ES HGSOC *versus* HC. In addition, increased MUC1 levels in circulating sEVs were confirmed by immunoassay (AUC = 0.840 for ES HGSOC *versus* HC; AUC = 0.860 for LS HGSOC *versus* HC, *p* < 0.05). In summary, our sEV proteomic analysis of ES HGSOC reveals exobiomarkers associated with early FT lesions, offering a promising avenue for detecting disease while it remains confined to the FT.

## Highlights

• LC–MS/MS custom spectral library–based circulating extracellular vesicle protein profile in high-grade serous ovarian cancer (HGSOC).
• Fallopian tube–lineage and serous tubal intraepithelial carcinoma–associated upregulated extracellular vesicle protein profile in HGSOC.
• Candidate diagnostic biomarkers: MYL6, GSTP1, TTYH3, PRDX6, MUC1, MYH14, and PTGS1.
• Combinatorial panel (MUC1, MYL6, TTYH3, and GSTP1) for detecting early-stage HGSOC.

## Graphical Abstract

**Figure:**
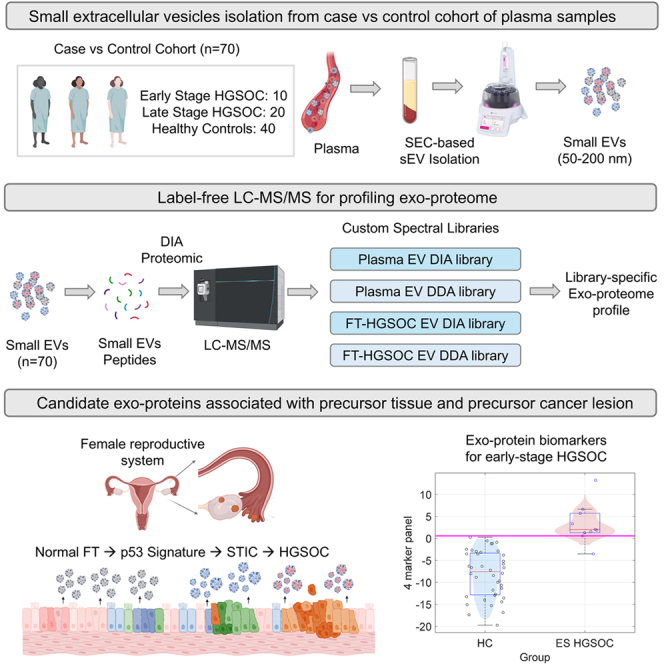

## In Brief

Small extracellular vesicles (sEVs) are emerging liquid biopsy biomarkers that carry proteins reflective of disease states. Using LC–MS/MS to profile plasma sEVs from a 70-sample case–control cohort, we identified circulating EV protein biomarkers that distinguish early-stage high-grade serous ovarian cancer from controls. These candidate biomarkers highlight the potential of sEV proteomics to enable minimally invasive detection of high-grade serous ovarian cancer while the disease remains confined to the fallopian tube.

## Introduction

Ovarian cancer is a collection of gynecological malignancies associated with the ovaries, fallopian tubes (FTs), and intraperitoneal cavity, with the highest case-to-fatality ratio (1). Often reported as a silent killer, these are frequently not diagnosed until the disease reaches an advanced stage, contributing to a poor 5-year survival rate of 49.7%. Current screening tests include gynecological evaluation with transvaginal ultrasound and molecular analysis of cancer antigen-125, which have low predictive value and have shown no significant beneficial effect on the mortality of ovarian cancer (2). As such, there is a critical unmet need to identify early detection strategies for the effective management of ovarian cancer.

Understanding the early events of the origin and development of ovarian cancer is fundamental in identifying novel diagnostic biomarkers for early detection. Studies have shown that the most common and fatal type of epithelial ovarian cancer is high-grade serous ovarian cancer (HGSOC), which originates from the FT (3, 4, 5). The tubal paradigm proposes that serous tubal intraepithelial carcinoma (STIC) present in the fimbriated end of the FT can become invasive and spread onto the peritoneal surface and ovary(s), giving rise to HGSOC (6). Following the model of progressive tumor development, epithelial cells in the FT acquire a *TP53* mutation(s), leading to STIC(s) and subsequently HGSOC. It is estimated that it may take 6 to 7 years to transition from active STIC to HGSOC, and dormant STIC may take up to 2 decades to develop into a carcinoma (5, 7). This timeline offers a significant window of opportunity to diagnose the disease early, while it is localized to the FT and potentially curative.

Early detection has been a key goal in cancer management. Efforts have been driven toward noninvasive detection of biomarkers using body fluids like blood, also known as a liquid biopsy. Extracellular vesicles (EVs), tiny lipid bilayer–delimited particles released by cells, are a new class of liquid biopsy biomarkers (8). The multianalyte cargo (protein, lipid, and nucleic acid) protected by lipid bilayer, high abundance in circulation, and universal secretion by all cell types make them an ideal candidate for biomarker development. EVs are heterogeneous with various subtypes based on their size and biogenesis pathways (9). Here, we focus on small extracellular vesicles (sEVs), with a size range of 50 to 200 nm. sEVs contain a cargo of selectively sorted biomolecules (proteins, lipids, and nucleic acids) that can mirror the physiological status of the cells of origin (10, 11, 12). sEVs play an active role in early pathogenesis and are secreted in circulation with cargo reflective of early malignancy and hence could be used as diagnostic biomarkers for early detection (13, 14, 15, 16). As such, several studies, including ours, have focused on exploring EV-based diagnostic biomarker development for early cancer detection (17, 18, 19, 20, 21, 22).

One of the major challenges in blood-based early-detection biomarkers is the extremely low abundance of secreted biomarkers in blood at the early stage (ES) of tumor development. Detecting circulating biomarkers shed by early lesions is limited by fundamental biological and mass transport barriers (8, 23). As most circulating EVs come from blood cells, the amount of tumor-derived EVs at the ES may represent as low as 0.1% of the total EV population (24, 25). As such, identifying tissue-lineage–specific EV biomarkers and precursor lesion–relevant EV biomarkers is crucial to capture these rare EV subpopulations. Although various studies have shown the potential of sEV-based biomarkers for various cancers, disease-specific exoprotein markers are still lacking. To address this potential challenge and limitation, in our previous studies, we have identified FT lineage–specific exoprotein biomarkers by carrying out a comprehensive proteomic profile of FT and HGSOC cells and tissue explant–derived sEV, with the potential of detecting HGSOC early while localized to the FT (22). In this study, using plasma samples from an archival case *versus* control cohort, we have further expanded exoprotein-based diagnostic biomarker discovery following a data-independent acquisition (DIA)–based LC–MS/MS with four custom spectral peptide libraries, including disease-specific spectral libraries with sEV peptides directly coming from the potential source of disease: FT and HGSOC cells and tissue explants.

Here, we present three notable approaches to identify candidate exoprotein-based diagnostic biomarkers in circulation associated with early cancer lesions (STIC present in the FT): (1) using disease-specific custom spectral libraries to identify exoproteome enriched from FT and HGSOC cell and tissue explants, (2) *in silico* data analysis to compare identified exoproteins to established FT–HGSOC core exoproteome and STIC tissue proteome, and (3) validation of exoprotein expression in normal FT tissue, FT tissue with STIC, HGSOC tissue, and FT and HGSOC cell line–derived sEVs. These candidate exoproteins can potentially reflect the earliest changes happening in the precursor lesion in FT epithelium that are reflected in circulating sEVs, providing a real-time spatiotemporal fingerprint for early detection of HGSOC.

## Experimental Procedures

### Human Samples

Deidentified plasma samples were obtained from the University of Kansas Medical Center Biospecimen Repository Core Facility following a protocol approved by the University of Kansas Medical Center’s Internal Review Board (HSC #5929). The studies have been performed in accordance with the ethical standards as laid down in the 1964 Declaration of Helsinki and its later amendments or comparable ethical standards. A case–control cohort of 70 archival patients was selected for the study, which includes 10 ES (FIGO [International Federation of Gynecology and Obstetrics] stage I and II) HGSOCs, 20 late-stage (LS; FIGO stage III and IV) HGSOCs, and 30 age and race-matched healthy controls (HCs), with an additional 10 HCs. The inclusion criteria for cases were pretreatment, presurgery, and samples taken at the time of diagnosis when the patient had active disease. The inclusion criteria for HCs were no current diagnosis of cancer, no prior history of cancer, and no chemotherapy or surgery related to cancer. Blood samples were collected in a vacutainer anticoagulant–treated tube (EDTA treated) and then centrifuged at 1300*g* for 10 min. The supernatant plasma was then stored in 1.0 ml Matrix tubes at −80 °C, with no freeze–thaw cycles occurring prior to EV purification. Time elapsed between sample collection and the current date at sample acquisitions ranges from ∼2 years to ∼12 years. Clinical information of patients is provided in Supplemental Table S1.

### Cell Culture

This study uses three immortalized human FT cell lines (FT240, FT194, and FT282) and three established HGSOC cell lines (OVCAR3, OVCAR8, and OVSAHO). FT cell lines were kindly provided by Dr Ronny Drapkin (University of Pennsylvania). FT cells were cultured with Dulbecco's modified Eagle's medium/F-12 (1:1) media without l-glutamine (Gibco, #21331020) supplemented with GlutaMAX (Gibco, #35050061) at a final concentration of 1×, 10% v/v fetal bovine serum (R&D Systems, #S11150) following EV depletion (spun at 100,000*g* at 4 °C for at least 18 h and filtered using a 0.2 μm filter), and 1% v/v penicillin–streptomycin (Gibco, #15140122) at 37 °C and 5% CO_2_. Ovarian cancer cell lines were cultured with RPMI 1640 medium (Gibco, #11875093) supplemented with 0.3% of 2.5 mg/ml insulin, 10% v/v fetal bovine serum following EV depletion, and 1% v/v penicillin–streptomycin at 37 °C and 5% CO_2_. Cell cultures were maintained exclusively in EV-depleted fetal bovine serum to avoid bovine EV contamination. Conditioned media from these cell lines were collected, and sEVs were isolated for validation experiments as previously described (22, 26).

### sEV Enrichment From Plasma

sEVs were enriched following an optimized size-exclusion chromatography (SEC)–based method. An IZON qEV1 70 nm column was used, and elution fractions were collected using an IZON automatic fraction collector (AFC) to improve reproducibility across sample size. Aliquots (1 ml) of previously banked plasma (EDTA) samples were centrifuged at 1500*g* for 10 min at 4 °C to remove cells, platelets, and apoptotic bodies. Supernatant was centrifuged at 10,000*g* for 10 min at 4 °C to remove larger particles (>1000 nm) and microvesicles. The supernatant was recovered to load into the IZON qEV1 column. The qEV1 column was first drained and washed with 13 ml PBS two times, as per the manufacturer's recommendation, and 1 ml plasma (supernatant after two centrifugation steps reported earlier) samples were loaded into the SEC column. Twenty fractions of 700 μl each were obtained. EV concentration and protein content in fractions 5 to 16 were quantified using nanoparticle tracking analysis (NTA) and Bradford assay, respectively, to identify sEV-rich fractions from protein-rich fractions. F7–F11 were selected as sEV-rich fractions, which translate to the following parameters in AFC: buffer volume: 4 ml (void volume that is discarded) and purified collection volume: F1–F5 (700 μl per fraction) (EV-rich volume that is collected). EV-rich fractions were pooled together (3.5 ml) and concentrated using an Amicon filter (Ultracel-10K Millipore, 10 K molecular weight cutoff, 4000*g*, 20 min, 4 °C) to a volume between 300 and 400 μl. Purified sEVs were stored at −80 °C until further use.

### sEV Characterization

sEVs were quantified using NTA. sEVs were diluted 100 to 300 times in PBS. Diluted sEVs (500 μl) were injected into the NanoSight LM10 system, and data were recorded using NTA software, version 2.3 (NanoSight Ltd), with the following settings: camera level 12, detection threshold 4, and temperature 20 °C. sEV protein content was quantified using the Bradford assay.

### Capillary-Based Western Blot to Validate Exoproteins

Expression of exoproteins was validated using a capillary-based simple Western assay (JESS, ProteinSimple). sEV at a concentration of 400 μg/ml or 600 μg/ml and cell lysates at a concentration of 600 μg/ml were used for the assay. The details of the antibodies used for the assay are provided in Supplemental Table S2. Based on the target protein of interest and its molecular weight, different separation modules (2–40 kDa, 12–230 kDa, and 44–400 kDa) were used with a capillary cartridge, which takes place in a fully automated system. A secondary anti-rabbit or anti-mouse detection module was used (based on the primary antibody) for detection following the manufacturer’s protocol. Blot images were taken using Compass software, version 6.0.0 (ProteinSimple) using High Dynamic Range 4.0, and contrast was manually adjusted for each set of samples.

### Transmission Electron Micrograph of sEV

Freshly thawed plasma-derived sEVs were diluted 1:1 in filtered PBS for transmission electron microscopy (TEM). sEVs (20 μl) were placed on carbon film–coated 300-mesh copper grids treated with glow discharge for 20 min. The grids were subsequently rinsed with six sequential droplets of distilled water, followed by staining for 5 s with 1% uranyl acetate. The grids were air-dried for 15 min and imaged using a JEOL JEM-1400 transmission electron microscope.

### Proteomics Analysis *Via* LC–MS/MS

#### Quality Control

An in-house digested lysate of the JJN-3 cell line is used as a control before and after each DIA experiment. The total number of proteins identified, the peak width (<0.3 min) measured from a particular MS1 signal (750.34 *m/z*), the intensity (>1e7) and retention time (20 ± 0.5 min) of the same signal, and the intensity of the MS2 total ion chromatogram (>2e8) for this precursor are all used to verify whether the UltiMate 3000 RSLCnano system and Exploris 480 (Thermo) are working properly. These measurements are compared with previous runs and have been monitored for consistency for >18 months. A check on the technical variability is done by injecting a pool of samples analyzed before, during, and after the experiment. This ensures the absence of drift or sudden degradation of the system during data collection. Samples are injected in a random order. Block randomization is employed. As an illustrative example, the order of the first 20 samples was GA12, GC9, GB5, GF3, GD3, GB1, GB12, GD1, GD10, GC5, GC7, GC3, GC2, GA3, GD11, GE7, GF1, GD12, GC8, and GD6.

#### Sample Preparation: Single-Pot, Solid Phase–Enhanced Sample Preparation 3 Method

Total protein from each sample was reduced, alkylated, and digested using single-pot, solid phase–enhanced sample preparation (27) with sequencing-grade modified porcine trypsin (Promega).

#### Data-Dependent Acquisition for Spectral Library Generation

A pool of all samples, after digestion, was made by combining 10% of each sample. Peptides were separated into 46 fractions on a 100 × 1.0 mm Acquity BEH C18 column (Waters) using an UltiMate 3000 UHPLC system (Thermo) with a 50 min gradient from 99:1 to 60:40 buffer A:B ratio under basic pH conditions and then consolidated into 18 superfractions.

Buffer A = 10 mM ammonium hydroxide, 0.5% acetonitrile in water

Buffer B = 10 mM ammonium hydroxide in acetonitrile

Each superfraction was then further separated by reversed-phase XSelect CSH C18 2.5 μm resin (Waters) on an in-line 150 × 0.075 mm column using an UltiMate 3000 RSLCnano system (Thermo). Peptides were eluted using a 60 min gradient from 98:2 to 65:35 buffer A:B ratio. Eluted peptides were ionized by electrospray (2.4 kV), followed by mass spectrometric analysis on an Orbitrap Eclipse Tribrid mass spectrometer (Thermo). Mass spectrometry (MS) data were acquired using the Fourier transform MS analyzer in profile mode at a resolution of 120,000 over a range of 375 to 1200 m/z. Following HCD activation, MS/MS data were acquired using the ion trap analyzer in centroid mode and normal mass range with a normalized collision energy of 30%.

Buffer A = 0.1% formic acid, 0.5% acetonitrile

Buffer B = 0.1% formic acid, 99.9% acetonitrile

#### DIA for Spectral Library Generation

A pool of all samples was made by combining 10% of each sample. Tryptic peptides were then separated by reversed-phase XSelect CSH C18 2.5 μm resin (Waters) on an in-line 150 × 0.075 mm column using an UltiMate 3000 RSLCnano system (Thermo). Peptides were eluted using a 60 min gradient from 98:2 to 65:35 buffer A:B ratio. Eluted peptides were ionized by electrospray (2.2 kV) followed by mass spectrometric analysis on an Orbitrap Exploris 480 mass spectrometer (Thermo Scientific). To assemble a chromatogram library, six gas-phase fractions were acquired on the Orbitrap Exploris with 4 *m/z* DIA spectra (4 *m/z* precursor isolation windows at 30,000 resolution, normalized automatic gain control [AGC] target 100%, maximum inject time 66 ms) using a staggered window pattern from narrow mass ranges using optimized window placements.

Buffer A = 0.1% formic acid, 0.5% acetonitrile

Buffer B = 0.1% formic acid, 99.9% acetonitrile

### Data Analysis for Spectral Library Generation

After data collection, raw files were converted to mzML files using ProteoWizard 3.0. ScaffoldDIA (Proteome software) was used to configure DIA library searches. Narrow-window DIA files were searched against the human-predicted spectral library from Prosit (2023/01 UniProt) with 10 ppm precursor and fragment ion tolerances to generate an empirically corrected chromatogram library with a peptide and protein false discovery rate (FDR) of 1%. Wide-window DIA files were subsequently searched against the generated empirically corrected chromatogram library.

Scaffold DIA 3.3.1 was used to search DIA–MS samples. Sequence database: a *Homo sapiens* database was downloaded from Uniprot.org (UP000005640_9606_Homo_sapiens, October 2022) and includes 20,311 entries. Spectral libraries were created by searching the data-dependent acquisition (DDA) samples collected on the Orbitrap Eclipse. The raw files were searched by MaxQuant (MQ) 2.0.3 against the sequence database listed previously. Trypsin was used to enzymatically create peptides. A maximum of two missed cleavages were allowed in the search.

Fixed modification: carbamidomethyl (C)

Variable modifications: oxidation (M); acetylation (protein N-term); and phosphorylation (STY)

Mass tolerance for precursor ions: MS/MS tolerance (Fourier transform MS) 20 ppm

Mass tolerance for fragment ions: MS/MS tolerance (ion trap MS) 0.5 Da

The standard list of contaminants provided by MQ was used and excluded.

Minimum score for unmodified peptides: 0

Minimum score for modified peptides: 40

FDR set to 1.0%.

### EV sample data acquisition

Tryptic peptides were trapped and eluted on 3.5 μm CSH C18 resin (Waters) (4 mm × 75 μm) and then separated by reversed-phase XSelect CSH C18 2.5 μm resin (Waters) on an in-line 150 × 0.075 mm column using an UltiMate 3000 RSLCnano system (Thermo). Peptides were eluted at a flow rate of 0.300 μL/min using a 60 min gradient from 98% buffer A:2% buffer B to 95:5 at 2.0 min to 80:20 at 39.0 min to 60:40 at 48.0 min to 10:90 at 49.0 min and hold until 53.0 min and then equilibrated back to 98:2 at 53.1 min until 60 min. Eluted peptides were ionized by electrospray (2.4 kV) through a heated capillary (275 °C), followed by data collection on an Orbitrap Exploris 480 mass spectrometer.

Buffer A = 0.1% formic acid, 0.5% acetonitrile

Buffer B = 0.1% formic acid, 99.9% acetonitrile

Precursor spectra were acquired with a scan from 385 to 1015 Th at a resolution set to 60,000 with 100% AGC, maximum time of 50 ms, and an RF parameter at 40%. DIA was configured on the Orbitrap 480 to acquire 50 × 12 Th isolation windows at 15,000 resolution, normalized AGC target 500%, and maximum injection time 40 ms. A second DIA was acquired in a staggered window (12 Th) pattern with optimized window placements.

### Data Analysis

Proteins were identified and quantified by searching the UniprotKB database restricted to *H. sapiens* (proteome ID: UP000005640_9606, October 2022) using MQ 2.0.3.1 (Max Planck Institute) label-free quantification with a parent ion tolerance of 2.5 ppm and a fragment ion tolerance of 0.5 Da. MQ output (msms.txt file) was used as an import to build a spectral library (.blib) using Skyline 22.2.0.255 and converted to a library (.dlib) using EncyclopeDIA (version 1.12.31). This empirically corrected library was used to search the DIA files collected by the Exploris 480, perform quantitative analysis, and obtain a comprehensive proteomic profile. Proteins were identified and quantified using EncyclopeDIA (28) and visualized with Scaffold DIA using 1% false discovery thresholds at both the protein and peptide levels. Protein MS2 exclusive intensity values were assessed for quality using ProteiNorm (29), and the data were normalized using the method of Cyclic Loess (30). Normalized intensity of proteins identified in each library is reported in Excel file in Supplemental Tables S3. Quantitative peptide reports are reported in Excel file in Supplemental Table S4. Supplemental Tables S5 and S6 include MQ evidence data with peptides identified by two DDA libraries, respectively. Supplemental Tables S7 and S8 contain the peptide quant report with peptides identified by two DIA (gas-phase fractionation [GPF]) libraries, respectively. The MS proteomics data have been deposited to the ProteomeXchange Consortium *via* the PRIDE partner repository with the dataset identifier PXD067228 and 10.6019/PXD067228 (31). The data include four .sdia files for four libraries, with the number of distinct peptides identified and protein sequence coverage percentage for each protein across 70 samples. “.sdia files” can be analyzed using a free Scaffold DIA Viewer software available at https://www.proteomesoftware.com/products/scaffold-dia.

Library-specific peptide identification and quantification of DIA–MS EV samples were analyzed using Scaffold DIA (3.3.1). DIA–MS data files were converted to mzML format using ProteoWizard (3.0.19254). Samples were searched against four different spectral libraries—two DDA libraries and two DIA libraries. Samples were aligned based on retention times and individually searched against specific libraries with a peptide mass tolerance of 25.0 ppm and a fragment mass tolerance of 25.0 ppm. For the DDA and the DIA spectral library search, fixed modifications considered were carbamidomethylation (C) 57.0214635 Da, nonterminal. Variable modifications considered were acetylation at the N-terminal 42.01056 Da, oxidation (M) 15.5549 Da, phosphorylation (STY), 79.966331 Da. The digestion enzyme was assumed to be trypsin with a maximum of one missed cleavage site allowed. Only peptides with charges in the range (2, 3) and length in the range (6-30) were considered. Peptides identified in each sample were filtered by Percolator (3.01.nightly-13-655e4c7-dirty) to achieve a maximum FDR of 0.01. Individual search results were combined, and peptide identifications were assigned posterior error probabilities and filtered to an FDR threshold of 0.01 by Percolator (3.01.nightly-13-655e4c7-dirty). Peptide quantification was performed by EncyclopeDIA (1.12.31). For each peptide, the eight highest quality fragment ions were selected for quantitation. Proteins that contained similar peptides and could not be differentiated based on MS/MS analysis were grouped to satisfy the principles of parsimony. Protein groups with a minimum of two identified peptides were thresholded to achieve a protein FDR less than 1.0%.

### Immunohistochemistry Staining

Unstained formalin-fixed paraffin-embedded slides of normal FT, FT with STIC lesions, and HGSOC tissues were obtained from the Biospecimen Repository Core Facility at the University of Kansas Medical Center. STIC lesions in FT were confirmed by board-certified clinical pathologist, Dr Wei Cui. Formalin-fixed paraffin-embedded tissues were baked at 60 °C for 30 min. Slides were then deparaffinized using xylene with 5 min incubation (two times). Slides were hydrated using a gradient of ethanol incubation—5 min at 100% ethanol (two times), 5 min at 90% ethanol, and 5 min at 70% ethanol. Slides were kept at PBS for 5 min before doing antigen retrieval. Antigen retrieval was done using citrate buffer and incubation in a pressure cooker. Citrate buffer solution (1x) was prepared by diluting citrate buffer, pH 6.0, 10x (#C9999-1000Ml) in autoclaved water. Citrate buffer (1x) was first preheated in a pressure cooker. Around 750 ml of autoclaved water was added to the pressure cooker. Two hundred milliliters of 1x citrate buffer was added to the slide holder and incubated in a pressure cooker at high pressure for 1 min. After preheating, slides were added to citrate buffer solution and incubated in a pressure cooker for 15 min at high pressure for antigen retrieval. After that, slides were allowed to cool down in citrate buffer solution at room temperature for 15 min. Slides were then incubated in 1x Tris-buffered saline with 0.1% Tween-20 solution for 15 min. Slides were then prepared for staining using the ImmPRESS polymer kit from Vector Lab (#MP-7801, #MP-7802). A border circling the tissue was prepared by a hydrophobic pen (ImmEdge Pen, #H-4000) and incubated with a few drops of BLOXALL blocking solution for 20 min in a humidified chamber. Slides were then washed with PBS for 5 min and incubated with normal horse serum (2.5%) for 30 min in a humidified chamber. Primary antibodies (Supplemental Table S2) were diluted in normal horse serum and added to tissue sections to cover the tissue completely and incubated overnight at 4 °C in a humidified chamber. After overnight incubation, slides were washed twice in PBS for 5 min and incubated with horseradish peroxidase anti-rabbit or anti-mouse IgG polymer reagent (based upon primary antibody) for 30 min at room temperature in a humidified chamber. Following this, slides were processed for reaction and hematoxylin stain. Slides were washed with PBS twice for 5 min. The 3,3′-diaminobenzidine reagent (chromogen and diluent) was mixed 1:1 right before addition to slides. 3,3′-Diaminobenzidine reagents were then added to the slide and left for reaction for 1 to 5 min, based on the target antigen, to develop color. After that, the reagent was discarded, and the slides were kept in PBS. For hematoxylin stain, slides were incubated with hematoxylin solution (Sigma, #GHS332-1L) for 5 min, dipped three times in acid alcohol (0.5% concentrated HCl in 70% ethanol), and recovered with the addition of dilute ammonia (0.2% of 30% ammonium hydroxide solution in deionized water). Slides were then kept in PBS. Before imaging, a few drops of Fluoromount-G (Invitrogen, #00-4958-02) were added to the slide, and a coverslip was added gently. After 1 h, when the slides were dry, they were imaged using Digital Slide Scanner ZEISS Axioscan 7. Slides were imaged with a 40X brightfield microscope. Images were exported with the following settings: Black 50, Gamma 0.45, White 200, and region of interest size with 17020 W and 11628 H.

### Development of a Combinatorial Exoprotein Panel for the Detection of ES HGSOC

A combinatorial panel of exoprotein markers among the seven candidate diagnostic biomarkers was selected using normalized MS data. To identify an optimal panel, we conducted a marker selection analysis using the Akaike Information Criterion (AIC) across all possible combinations of the seven markers (120 models in total). The model that minimized the AIC was selected. For comparison, we also tested a naive sum of all seven markers, which gave an inferior area under the curve (AUC) (0.9135). After model selection, a four-marker panel, comprising MUC1, MYL6, TTYH3, and GSTP1, achieved an AUC of 0.9750. The logistic regression model for this panel is:

-427.13+53.401×log(MUC1)+16.365×log(MYL6)+26.481×log(TTYH3)+49.018×log(GSTP1)

where the first term could also be omitted without affecting the AUC value. It is sourced from the intercept of the underlying logistic regression model.

### MUC1 ELISA

Twenty plasma-derived sEV samples (from the discovery cohort) isolated from SEC were used to validate expression of MUC1 exoproteins using ELISA following the manufacturer's recommended protocol (Invitrogen, #EHMUC1). Samples include 5 ES HGSOC, 5 LS HGSOC, and 10 HCs. Plasma-derived sEVs (0.5 μg) were lysed using 0.1% Triton X-100 (Sigma, #X100-100ML) in 1X protease phosphatase inhibitor solution (Thermo Scientific, #1862209, 100X) with a final volume of 100 μl in PBS. The solution was bath sonicated for 20 min. Lysed EVs (100 μl) were added to ELISA wells with three technical replicates. MUC1 standards (100 μl; mU/ml) were added as well for the standard curve. Samples and standards were sealed and incubated for 2.5 h at room temperature with gentle shaking (250 rpm). Plates were then washed five times with 200 μl of wash buffer. After each wash, plates were gently tapped and soaked against a Kimwipe for complete removal of liquid. Antibody–biotin conjugate (100 μl) was added and incubated for 1 h at room temperature with gentle shaking. Following incubation, plates were washed five times with 200 μl of wash buffer. Streptavidin–horseradish peroxidase solution (100 μl) was added and incubated for 45 min at room temperature with gentle shaking. Following incubation, plates were washed five times with 200 μl of wash buffer. 3,3′,5,5′-Tetramethylbenzidine substrate (100 μl) was added and incubated for 30 min at room temperature in the dark with gentle shaking. After incubation, 50 μl of stop solution was added to the wells, gently mixed, and the absorbance was measured at 450 nm. Two standard curves were made using a linear scale of MUC1 concentration (mU/ml) and a log_10_ scale of MUC1 concentration (mU/ml). MUC1 concentration of sEV was identified using the equation of a standard curve.

### Experimental Design and Statistical Rationale

Experiments were designed with at least three biological or technical replicates for statistical analysis. For proteomic analysis, 10 biological replicates were used for ES HGSOC, 20 for LS HGSOC, and 40 for HC. For EV characterization *via* NTA, five technical replicates were used. For JESS-based Western blot analysis, three biological replicates for each cell line, FT or HGSOC, were used. For TEM analysis, three biological replicates were used for the HGSOC and HC cohorts. For immunohistochemistry (IHC)-based validation studies, three biological replicates were used for FT, FT with STIC, and HGSOC tissue. For the ELISA-based validation study, 5 biological replicates for ES HGSOC, 5 for LS HGSOC, and 10 for HC controls were used, with three technical replicates per sample. Each *Experimental Procedures* section provides a detailed experimental design and corresponding analysis. Differentially expressed proteins were identified using an unpaired *t* test with cutoffs of *p* < 0.05 and |log_2_-fold change [FC]| >1. For quality control, exoproteins with fewer than eight observations in any of the three groups: ES HGSOC (n = 10), LS HGSOC (n = 20), and HC (n = 40) were excluded. A minimum sample size of eight per group allows detection of effect sizes ≥1.5 with adequate power (∼0.77) using a nonparametric empirical area under the receiver operating characteristic curve and ∼0.80 using the parametric *t* test. Smaller sample sizes would be insufficient to reliably detect such effect sizes.

Heatmaps were generated using the ComplexHeatmap package (version 2.24.1) in R (32). Normalized expression values for differentially expressed proteins (*p* < 0.05, |log2(FC)| >1) were extracted from the raw dataset. Z-score normalization was applied to each protein (row wise) across all samples. Samples were ordered by group (healthy, ES HGSOC, and LS HGSOC). Pathway analysis was carried out using ShinyGO 0.82 with an FDR cutoff of 0.05 (33).

Receiver operating characteristic curves of the ELISA result were made using GraphPad Prism following the Wilson/Brown method with a 95% confidence interval. Pan-cancer heatmap showing differential expression of candidate genes in tumor *versus* normal tissue was created from “TNMplot: differential gene expression analysis in Tumor, Normal, and Metastatic tissues (tnmplot.com)” (34).

## Results

### Enrichment and Characterization of sEVs From Clinical Samples

**Fig. 1:**
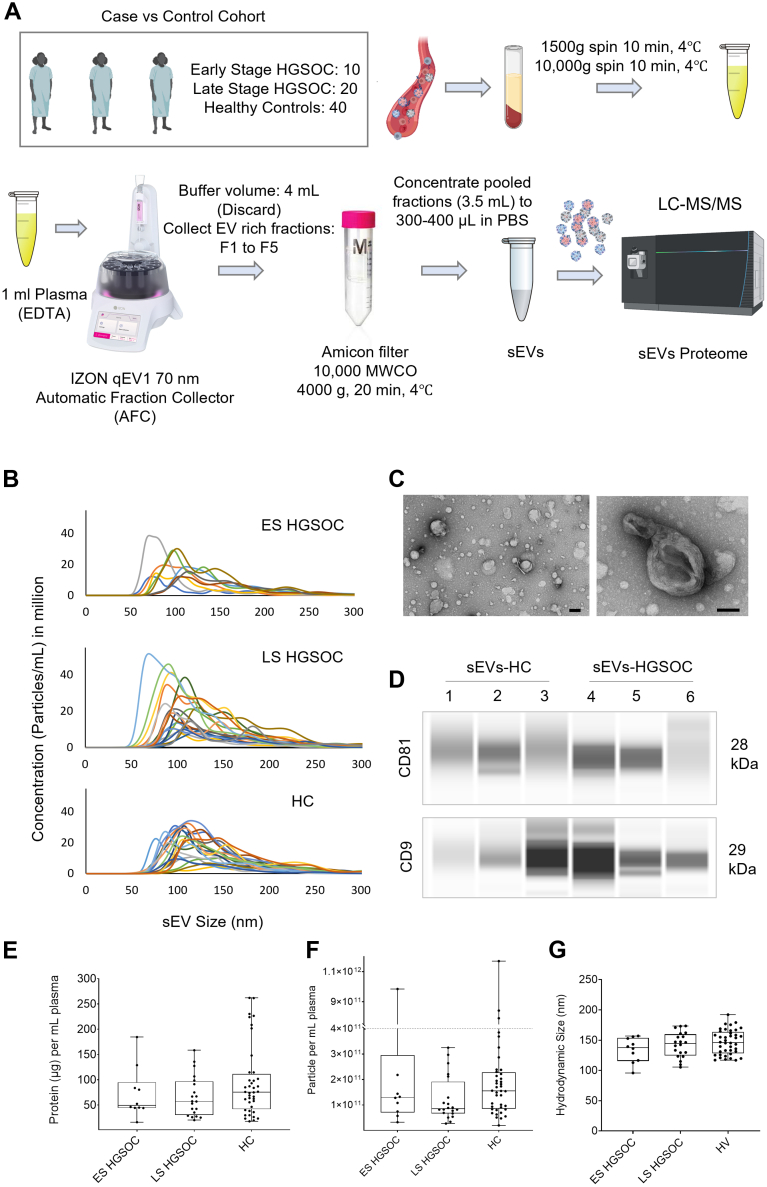
**Isolation and characterization of small extracellular vesicles (sEVs) from case *versus* control cohort of plasma samples.***A*, case *versus* control cohort of 70 plasma samples, including 10 early-stage, 20 late-stage high-grade serous ovarian cancer (HGSOC), and 40 healthy controls (HCs). Schematic showing size-exclusion chromatography for isolation of sEVs. *B*, size distribution profile of sEVs in 70 plasma samples calculated using nanoparticle tracking analysis (NTA). Each graph represents an sEV sample with five replicate measurements. *C*, representative transmission electron micrograph of the sEV sample at different magnifications (25,000X, the scale bar represents 100 nm) shows the morphological features of sEVs. *D*, molecular characterization of representative six plasma sEV samples showing the presence of EV marker proteins CD9 and CD81, quantified by capillary Western blot (JESS). *E*, protein quantification of sEV samples *via* Bradford assay. *F*, particle quantification of sEV samples *via* NTA. *G*, hydrodynamic size of sEV samples *via* NTA.

A case *versus* control cohort of 70 plasma samples was established, comprising 10 ES (ES, stage I and II) HGSOC, 20 LS (stage III and IV) HGSOC, and 40 age- and race-matched HCs to discover sEV-associated diagnostic protein biomarkers. sEVs were enriched from 70 plasma samples using an optimized SEC-based method (Fig. 1*A* & Supplemental Fig. S1*A*). SEC-based sEV isolation method was optimized for maximum EV yield with minimum soluble impurities (lipoproteins, albumin, and protein aggregates). For the purpose, 1 ml of processed plasma samples (see the *Experimental Procedures* section) was run through an IZON qEV1 70 nm column using an IZON AFC. Twenty fractions (F1–F20) of 700 μl volume per fraction were collected. Protein content and particle number were quantified for 10 fractions: F5 to F16. Particle data show that EV starts to elute from F7 and beyond, whereas protein content starts to increase significantly from F12 and beyond (Supplemental Fig. S1, *B* and *C*). This trend was similar in the two samples tested: 1 ml of HC plasma and HGSOC plasma. The marked increase of protein content in F12 and beyond suggests elution of soluble impurities from this fraction. Based on this dataset, fractions F7–F11 were designated as sEV enriched and pooled for the sEV isolation protocol. To translate this into AFC, the initial fractions with no particles are designated as buffer volume, which is set as 4 ml. Then, five AFC fractions (AFC F1–F5) are collected, which are equivalent to F7–F11 of the total fractions. The purity of these fractions was further assessed by calculating the particles-to-protein ratio. The particle-to-protein ratio was highest in fractions F1 to F5 (equivalent to F7–F11 in total fractions), further supporting that these fractions are enriched in sEVs with minimal soluble impurities (Supplemental Fig. S1, *D* and *E*). Following optimization, the SEC method for sEV isolation was established in the AFC with the following settings: 4 ml buffer volume discarded, collection of AFC fractions F1 to F5, with 700 μl volume per fraction. All 70 sEV samples were subsequently enriched using this established method.

sEV particle count and protein content were characterized by NTA and Bradford assay, respectively. NTA shows a size distribution profile of ∼50 to 250 nm across three cohorts (Fig. 1*B*). The surface morphology of sEV was characterized by TEM, which shows characteristic round, spherical, and cup-shaped morphology (Fig. 1*C*, Supplemental Fig. S2*A*). Molecular characterization of sEV was performed using capillary Western blot (JESS), which showed the presence of two common EV-associated tetraspanins, CD9 and CD81, in six representative samples (Fig. 1*D* & Supplemental Fig. S2*B*). The total protein amount, EV number, and mean hydrodynamic size of sEV were analyzed across three cohorts. Across 70 cases, the total protein content of 15 to 248 μg, total EV number of 1.96 × 10^10^ to 1.17 × 10^11^, and a mean size range of 95 to 192 nm were observed in 1 ml plasma samples (Fig. 1, *E*–*G*). There was no significant difference between sEV number, protein yield, and size between sEV from three different cohorts: ES HGSOC, LS HGSOC, and HC. Furthermore, we analyzed the particle-to-protein ratio across 70 samples, which showed a similar trend to particle number and protein amount, with no significant changes across three cohorts (Supplemental Fig. S2*C*). We observed a weak but statistically significant correlation between sEV particle number and protein content, with a Pearson's correlation value of 0.3245 (*p* < 0.05) across 70 samples (Supplemental Fig. S2*D*). In summary, we developed a semiautomated SEC-based protocol for reproducible enrichment of plasma sEVs and characterized their size, morphology, and cargo. Our data show wide variation in circulating sEV abundance across patients in each cohort, and the variation was similar in all three cohorts, suggesting that circulating sEV abundance varies largely across patients.

### Proteomic Characterization of Circulating sEVs Using LC–MS/MS

**Fig. 2:**
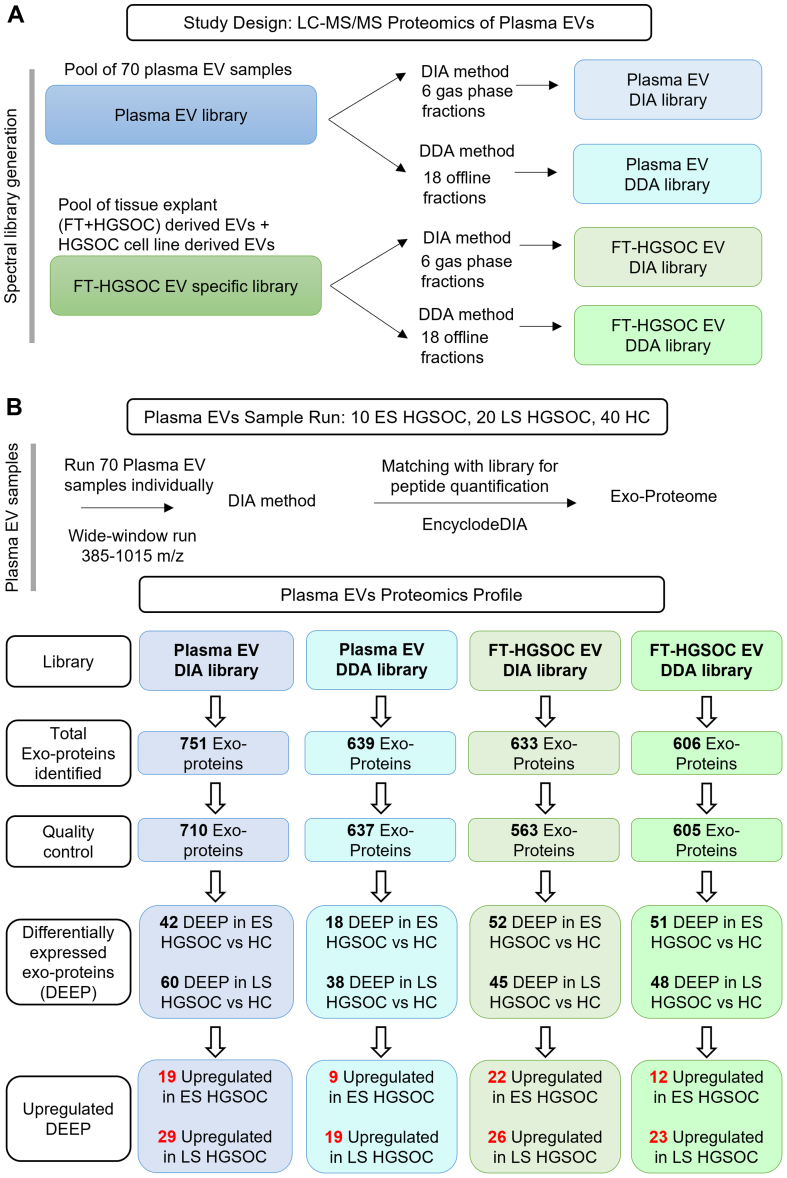
**LC–MS/MS study design and identification of plasma exoproteome.***A*, study design for LC–MS/MS plasma small extracellular vesicle (sEV) using label-free quantification. Four spectral libraries were generated from two sample types: pooled plasma sEVs and pooled FT–HGSOC sEVs, and two different methods of spectral library generation: data-dependent acquisition (DDA) and data-independent acquisition (DIA). The pooled plasma sEV sample was prepared from 70 individual plasma sEV samples, whereas the pooled FT–HGSOC sEV was prepared from sEVs derived from FT and HGSOC tissue explants and cell cultures. *B*, 70 individual plasma sEV samples were then analyzed using the DIA method and searched against four spectral libraries for protein identification and quantification. Across all samples and libraries, a total of 1078 sEV proteins (*i.e.*, exoproteins) were identified. For each library, identified exoproteins underwent quality control and differentially expressed exoproteins (DEEP) were identified (unpaired *t* test, log_2_FC >1, *p* < 0.05). For quality control, exoproteins with less than eight observations in at least one of the three groups: ES HGSOC (n = 10), LS HGSOC (n = 20), and HC (n = 40) were excluded from further analysis. ES, early stage; FT, fallopian tube; HC, healthy control; HGSOC, high-grade serous ovarian cancer; LS, late stage.

sEV proteome (exoproteome) was established using label-free DIA–based LC–MS/MS with a customized peptide library. In addition to the standard, GPF library created with pooled sEV samples (n = 70), we designed an FT/ovarian-specific library (FT-HGSOC library), which consisted of a pooled sEVs from (1) FT tissue explants, (2) HGSOC tissue explants, and (3) HGSOC cell lines. We have previously characterized sEV from these samples (22). Spectral peptide libraries were generated using both the DDA method with 18 offline fractions and the DIA method with 6 GPFs, resulting in 4 separate spectral libraries (Fig. 2*A*). Following the customized library generation, each sEV sample was run using the DIA method, and the identified peptides were matched with each of the four libraries separately for protein identification and quantitative analysis. The inclusion of four different customized libraries adds to the sensitivity and quantity of exoproteins discovered in this study. FT-HGSOC sEV library is enriched in peptides directly from the FT/ovarian source. Hence, it will help to aid in peptide identification in plasma sEV that are from the FT/ovarian source, which otherwise may not be detected in the plasma sEV pooled library because of low abundance.

MS identified 1078 exoproteins across four libraries, of which 325 were common to all libraries (Supplemental Fig. S3*A*). Among the 1078 exoproteins, 102 were commonly associated with EVs as reported by the MISEV 2021 guideline (35). Of these, 14 exoproteins were in category 1 (transmembrane or glycosylphosphatidylinositol–anchored proteins associated with plasma membrane or endosomes), 25 exoproteins in category 2 (cytosolic proteins in EVs), 20 exoproteins in category 3 (major components of non-EV coisolated structures), 4 exoproteins in category 4 (transmembrane, lipid-bound, and soluble proteins with intracellular compartments other than plasma membrane or endosomes), and 39 exoproteins in category 5 (secreted proteins recovered with EVs). These findings suggest the robustness of the enrichment and proteomics workflow (Supplemental Fig. S3, *B*–*F*).

Figure 2*B* shows the number of exoproteins identified in each library, ranging from 606 to 751, and illustrates the pipeline to sort exoproteins, which are more abundant in cases *versus* controls. Exoproteins detected in fewer than eight samples in any cohort were excluded during the quality control to ensure sufficient statistical power. Differentially expressed exoproteins in cases *versus* controls were then identified using an unpaired *t* test with parameters of |log_2_-FC| >1 and *p* < 0.05. Exoproteins were further ranked by AUC value to identify the top-performing candidates (Supplemental Fig. S4). For the biomarker discovery approach, upregulated differentially expressed exoproteins (log_2_-FC >1, *p* < 0.05) were prioritized. This pipeline yielded a set of 9 to 22 upregulated exoproteins in ES HGSOC *versus* HCs and a set of 19 to 29 upregulated exoproteins in LS HGSOC *versus* HCs.

**Fig. 3:**
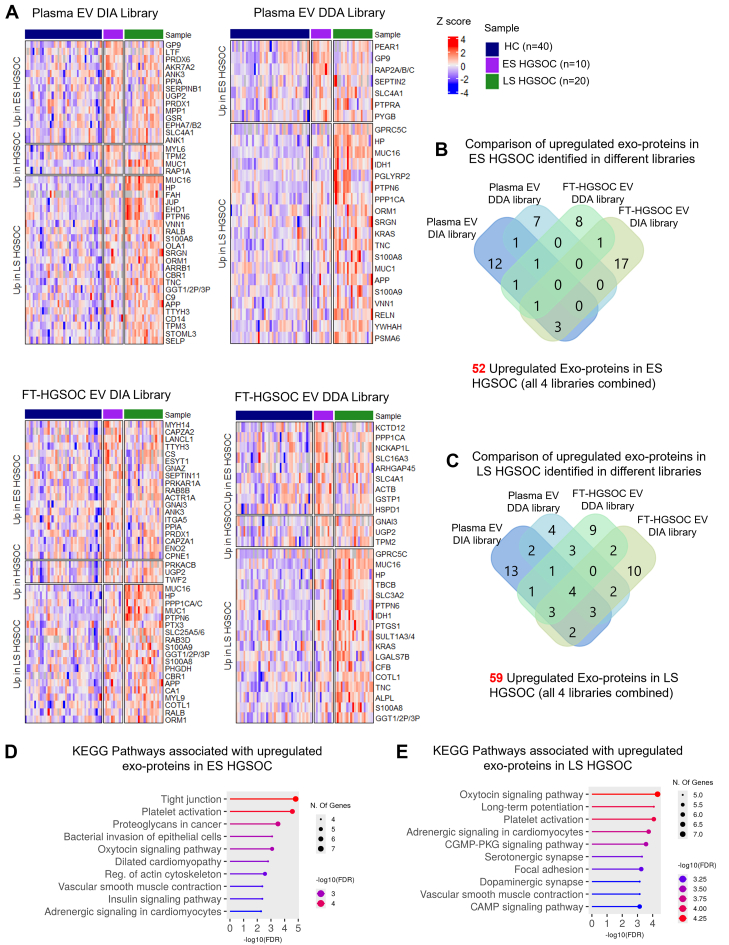
**Identification of upregulated exoproteins in early-stage (ES) and late-stage (LS) HGSOC.***A*, heatmap showing upregulated exoproteins in ES and LS HGSOC compared with healthy controls (HCs) across four different libraries. Each column represents a single sample. Protein expression values are displayed as row-wise z-scores. Missing values are shown in *gray*. Sample groups are color-coded: HC (*blue*), ES HGSOC (*purple*), and LS HGSOC (*green*). *B* and *C*, comparison of upregulated exoproteins across all four libraries identifies 52 upregulated exoproteins in ES HGSOC and 59 exoproteins in LS HGSOC, respectively. Comparison reveals some exoproteins are upregulated exclusively in given libraries, whereas some are shared between libraries. *D* and *E*, upregulated KEGG pathways associated with 52 upregulated exoproteins in ES HGSOC and 59 exoproteins in LS HGSOC, respectively. Pathway analysis was done using ShinyGO 0.82. HGSOC, high-grade serous ovarian cancer; KEGG, Kyoto Encyclopedia of Genes and Genomes.

A heatmap of upregulated exoproteins in ES HGSOC *versus* HC and LS HGSOC *versus* HC is shown in Figure 3*A*. Comparison of upregulated exoproteins across four spectral libraries highlights both shared and unique exoproteins (Fig. 3, *B* and *C*). Across all libraries, 52 upregulated exoproteins were identified in ES HGSOC *versus* HC, and 59 exoproteins were identified in LS HGSOC *versus* HC, combined (Supplemental Table S9). Pathway analysis of these exoproteins shows upregulation of various biological pathways, including platelet activation in both ES and LS cases, which is known to be associated with ovarian cancer pathology (Fig. 3, *D* and *E*) (36).

### Identification of Candidate Exoprotein Diagnostic Biomarkers Based upon FT-Cell Lineage and Early-Cancer Lesion

STIC lesions in the FT are precursors for most HGSOCs. To identify candidate exoproteins of interest relevant to the ES of disease progression, we applied these three criteria: (1) FT lineage, (2) STIC association, and (3) membrane expression. The latter was prioritized based on our previous work of detecting EV membrane proteins using microfluidic platforms (20, 22, 37, 38).

**Fig. 4:**
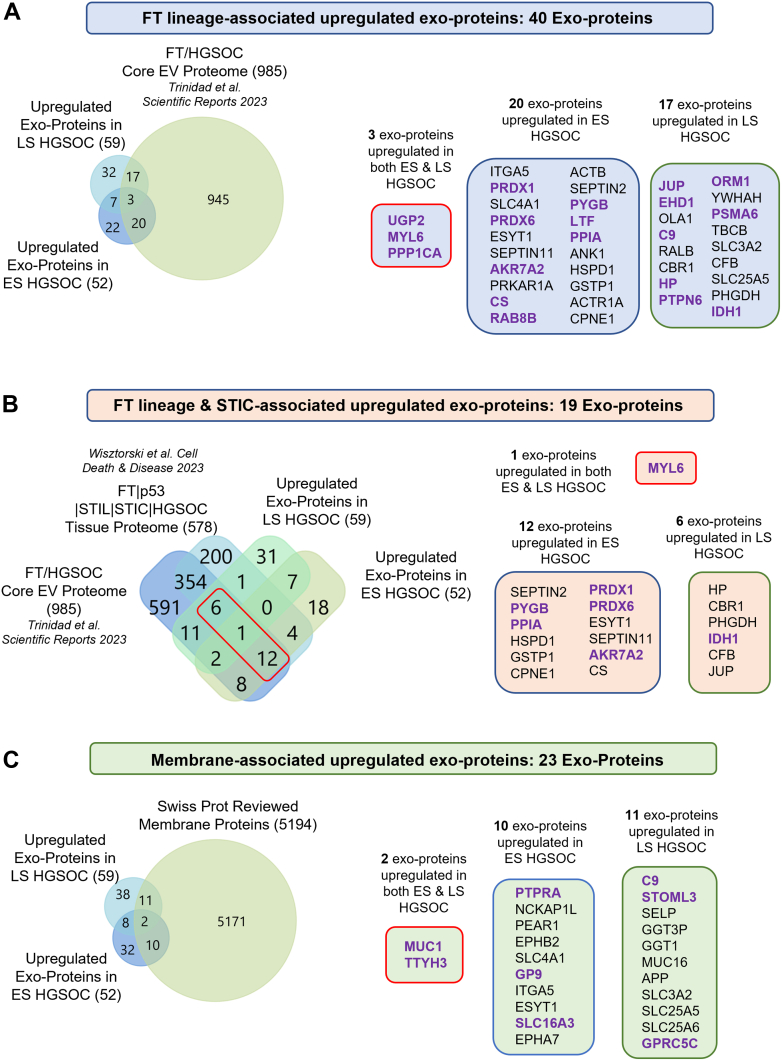
**Candidate exoprotein diagnostic biomarkers for early-stage (ES) and late-stage (LS) HGSOC sorted based upon FT-lineage, STIC association, and surface expression.***A*, upregulated exoproteins in ES and LS HGSOC were compared with the FT–HGSOC core EV proteome dataset (22), identifying 40 exoproteins that are of potential FT lineage. *B*, upregulated exoproteins in ES and LS HGSOC were compared with both the FT–HGSOC core EV proteome dataset (22) and tissue proteome conserved between FT, p53 signature, STIL, STIC, and HGSOC (39) to sort for 19 exoproteins that are potential FT lineage and expressed in STIC. *C*, upregulated exoproteins in ES and LS HGSOC were compared against the SwissProt Reviewed Membrane proteins database identifying 23 membrane-associated exoproteins. The multitiered sorting approach helps to select candidate exoproteins relevant to ovarian cancer pathology and supports in identifying potential exoprotein-based diagnostic biomarkers for EV capture and detection assays. Proteins in *purple* indicate those ranked in the top 10 list by AUC value for discriminating cases *versus* controls. AUC, area under the curve; EV, extracellular vesicle; FT, fallopian tube; HGSOC, high-grade serous ovarian cancer; STIC, serous tubal intraepithelial carcinoma; STIL, serous tubal intraepithelial lesion.

We compared the circulating exoproteome from this study with previously reported exoprotein and tissue protein datasets. As a reference, we used our previously defined FT/HGSOC core exoproteome dataset (comprising proteins common between FT and HGSOC cell and tissue explant–derived EVs) (22). Fifty-two upregulated exoproteins in ES HGSOC and 59 upregulated exoproteins in LS HGSOC were evaluated against the 985 exoproteins in the FT/HGSOC core dataset. This analysis identified 40 shared exoproteins, designated as FT lineage–associated exoproteins upregulated in HGSOC cases (Fig. 4*A*). Among these, three exoproteins (MYL6, UGP2, and PPP1CA) were upregulated in both ES and LS HGSOC, whereas 20 exoproteins were specific to ES and 17 to LS cases. Next, we incorporated a tissue proteome dataset in our comparison, which includes conserved proteins across the progression of FT to HGSOC: common 578 tissue proteins across FT, p53 signature, STIL (serous tubal intraepithelial lesion), STIC, and HGSOC tissue, as reported by Wisztorski *et al*. (39). Comparison with our exoproteome identified 19 common proteins, which we designated as FT lineage and STIC-associated exoproteins upregulated in HGSOC cases (Fig. 4*B*). Of these, MYL6 was upregulated in sEVs in both ES and LS HGSOC patient samples; 12 were specific to ES and 6 to LS cases.

We further compared our circulating exoprotein dataset with the membrane protein database (SwissProt reviewed membrane proteins), identifying 23 membrane-associated exoproteins upregulated in HGSOC (Fig. 4*C*). Among these, MUC1 and TTYH3 were upregulated in both ES and LS HGSOC; 10 were specific to ES and 11 to LS cases. These membrane exoproteins are candidates for immunocapture-based EV enrichment and detection platforms.

In summary, we identified 40 exoproteins of FT lineage, 19 of FT lineage and STIC association, and 23 membrane-expressed exoproteins upregulated in circulating sEVs from HGSOC cases. Exoproteins with high AUC values (top 10 in the group) are in purple text, indicating potential high-performance markers to discriminate between case *versus* control (Fig. 4 & Supplemental Fig. S4). These candidate circulating exoproteins are relevant to HGSOC pathogenesis and may be applied in a capture and detection platform for diagnostic biomarker discovery.

### Validation of Candidate Exoprotein Diagnostic Biomarker in Precursor Lesion and Cell Line–Derived EVs

We selected seven candidate exoprotein diagnostic biomarkers for validation based on FT lineage, STIC association, surface expression, and case–control discriminatory power (high AUC value and tissue expression in case *versus* control). Based on these criteria, MYL6, GSTP1, PRDX6, MUC1, TTYH3, MYH14, and PTGS1 were prioritized for validation (Supplemental Table S10). MYL6, GSTP1, and PRDX6 are FT-lineage and STIC-associated exoproteins elevated in ES HGSOC *versus* HC. MUC1 and TTYH3 are membrane exoproteins enriched in ES HGSOC *versus* HC. MYH14 and PTGS1 are upregulated in ES *versus* HC and LS *versus* HC, respectively, with high discriminatory power (high AUC value and tissue expression in case *versus* control).

**Fig. 5:**
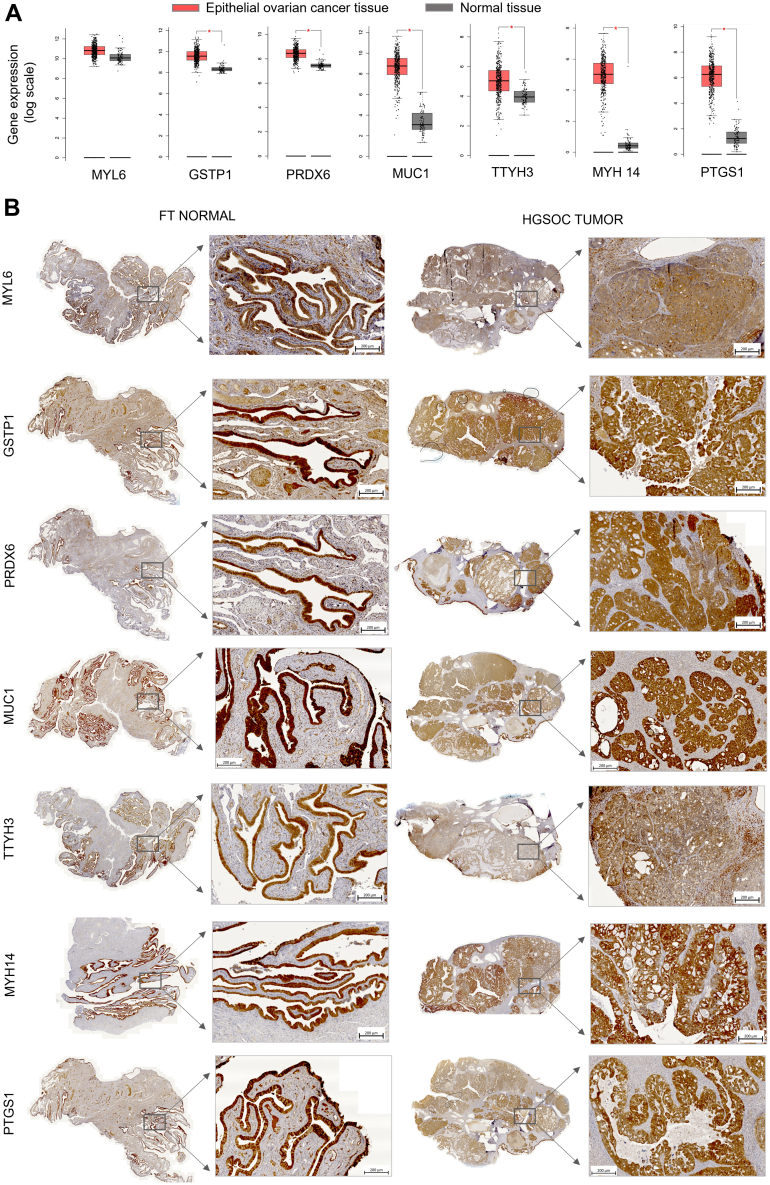
**Expression patterns of seven candidate exoproteins in fallopian tube (FT) and high-grade serous ovarian cancer (HGSOC) tissue.** Seven exoproteins were selected for validation based on FT lineage, STIC association, membrane expression, and differential gene expression profile in HGSOC tissue compared with normal ovarian tissue. *A*, gene expression profiles of the seven exoproteins in epithelial ovarian cancer tissue compared with normal tissue, showing elevated expression in HGSOC (∗logFC >1). Box plots represent mRNA expression from 426 epithelial ovarian cancer tissues and 88 normal tissues, generated using the Gene Expression Profiling Interactive Analysis (GEPIA) (http://gepia.cancer-pku.cn/), an open-access database based on The Cancer Genome Atlas (TCGA) and Genotype-Tissue Expression (GTEx) data. *B*, IHC staining of seven exoproteins in representative FT and HGSOC tissue samples using formalin-fixed paraffin-embedded slides. FC, fold change; IHC, immunohistochemistry; STIC, serous tubal intraepithelial carcinoma.

We evaluated the gene expression profile (mRNA profile) of these seven exoproteins using The Cancer Genome Atlas and Genotype-Tissue Expression (GTEx) datasets, observing higher gene expression in epithelial ovarian cancer tissue (n = 426) compared with normal ovarian tissue (n = 88) (Fig. 5*A*). Note that normal tissue data from the GTEx dataset represent bulk ovary containing heterogeneous cell types, whereas epithelial ovarian cancer samples are enriched in epithelial cell type, which may introduce potential confounding factors. Six of seven markers (except MYL6) showed statistically significant overexpression in epithelial ovarian cancer compared with normal ovarian tissue (log_2_FC >1). Protein expression was validated by IHC in normal FT epithelium and HGSOC tissues, confirming expression of all seven proteins (Fig. 5*B*, Supplemental Figs. S5 and S6).

**Fig. 6:**
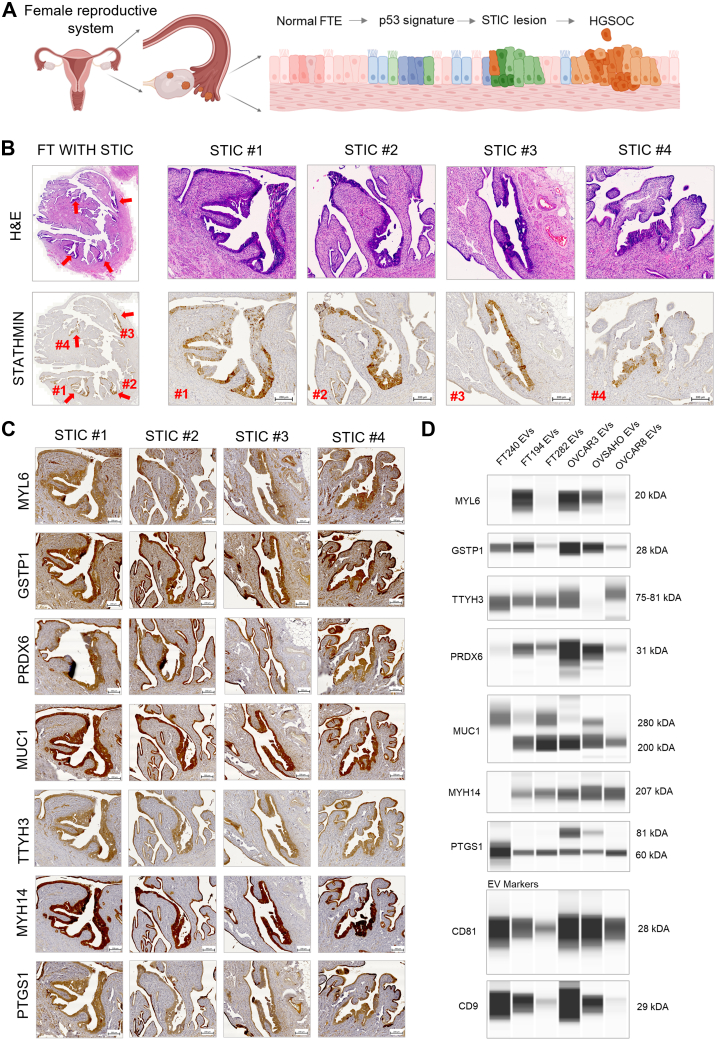
**Expression of seven candidate exoproteins in STIC lesion and cell culture–derived small extracellular vesicles (sEVs).***A*, schematic showing the pathology of HGSOC, from the normal fallopian tube (FT), p53 signature, STIC lesion, and HGSOC. *B*, H&E staining of the FT tube with various STIC lesions as confirmed by the pathologist. STIC lesion was further confirmed by staining STIC marker protein STATHMIN in formalin-fixed paraffin-embedded slides. Four major STIC lesions were identified, as denoted by *red arrows* and numbers #1, #2, #3, and #4. *C*, expression of seven exoproteins in STIC lesions using IHC on formalin-fixed paraffin-embedded slides, confirming the expression of these seven exoproteins in STIC lesions. *D*, evaluation of exoprotein expression in three FT tubes and three HGSOC cell line–derived sEVs using capillary-based Western blot (JESS). CD81 and CD9 are EV marker proteins. HGSOC, high-grade serous ovarian cancer; IHC, immunohistochemistry; STIC, serous tubal intraepithelial carcinoma.

Next, we validated expression of candidate exoproteins in (1) STIC tissue, the precursor lesion of HGSOC (Fig. 6*A*) and (2) sEVs from FT and HGSOC cell lines using IHC and capillary-based Western blot. A representative IHC image of FT epithelium with four major STIC lesions, validated by a pathologist, is shown in Figure 6*B*. STIC lesions were further validated by Stathmin, a marker specific to STIC (40). IHC confirmed the expression of all seven candidate exoproteins in the STIC lesions (Fig. 6*C* and Supplemental Fig. S7), supporting their potential cancer relevance.

We also assessed EVs from three different normal FT immortalized cell lines (FT240, FT194, and FT282) and three different HGSOC cell lines (OVCAR3, OVSAHO, and OVCAR8) using capillary Western blot (Fig. 6*D* and Supplemental Fig. S8), with cell lysates as positive controls (Supplemental Figs. S9 and S10). Western blot confirmed expression of exoproteins in most cell line–derived EVs, from both FT and ovarian cancer cells, further supporting their FT/HGSOC origin and their elevated levels in the circulation of ES HGSOC patients.

### Development of a Combinatorial Panel of Exoprotein Diagnostic Biomarker and Validation of Candidate Exoproteins in Circulating EVs

**Fig. 7:**
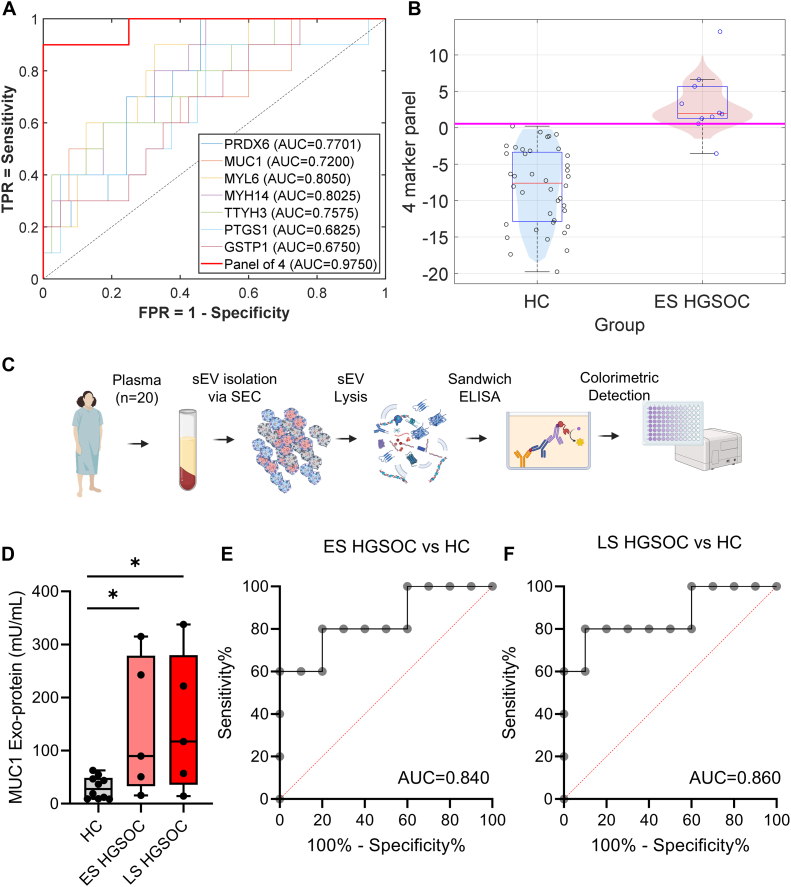
**Development of a combinatorial exoprotein diagnostic biomarker panel and assessment of MUC1 exoprotein in plasma-derived extracellular vesicles (EVs).***A*, ROC–AUC analysis of the seven candidate exoprotein biomarkers and identification of an optimal four-marker combinatorial panel based on Akaike Information Criterion (AIC) model selection. *B*, the optimized Youden-based cutoff demonstrates a sensitivity of 90% at a specificity of 100%, highlighting the strong discriminatory performance of the four-marker exoprotein panel for distinguishing early-stage (ES) HGSOC from healthy controls (HCs). The Youden-based cutoff (*magenta line*) corresponds to a specificity of 1.000 and to a sensitivity of 0.9000. *C*, schematic of ELISA-based colorimetric detection of MUC1 exoproteins from plasma-derived EVs. *D*, distribution of MUC1 concentration across samples, showing significantly higher concentrations in ES HGSOC and late-stage (LS) HGSOC compared with HC (∗*p* < 0.05, two-tailed unpaired *t* test). *E*, area under the receiver operating characteristic curves showing sensitivity and specificity of MUC1 exoproteins to discriminate ES HGSOC *versus* HC, and *F*, LS HGSOC *versus* HC. AUC, area under the curve; HGSOC, high-grade serous ovarian cancer.

We employed AIC to select the best model for combinatorial marker selection that can have superior discriminatory characteristics to detect ES HGSOC. One hundred twenty different models were applied to the normalized intensity data from LC–MS/MS, and the one that minimized the AIC was selected (see the *Experimental Procedures* section). Based on the selected model, a four-marker panel comprising exoproteins, MUC1, MYL6, TTYH3, and GSTP1, showed an AUC of 0.975, with a sensitivity of 90% at 95% specificity (Fig. 7*A* & Supplemental Table S11). The optimized Youden-based cutoff shows sensitivity of 90% at specificity of 100%, showing superior discriminatory characteristics of the combinatorial exoprotein panel to detect ES HGSOC from HC (Fig. 7*B*). Pathway analysis shows that these markers participate in distinct and nonredundant biological pathways, covering metabolism (GSTP1), signaling/adhesion (MUC1), cytoskeleton/mechanics (MYL6), and ion channel–mediated migration and mitogen-activated protein kinase signaling (TTYH3), making the panel biologically complementary and, thus, potentially more robust and relevant for early ovarian cancer detection (Supplemental Table S12). Pathway analysis using ConsensusPathDB confirmed that these four exoproteins map predominantly to separate functional modules rather than a single shared pathway, further supporting the biological complementarity of the panel (Supplemental Table S13).

Based on their abundance in circulating EVs, we further validated the presence of MUC1 exoproteins in plasma-derived sEVs from 20 representative samples (10 HCs, 5 ES HGSOCs, and 5 LS HGSOCs) using ELISA (Fig. 7*C*). MUC1 concentrations (mU/ml) were calculated from absorbance values using a standard curve (Supplemental Fig. S11*A*). The measured range of MUC1 exoproteins was 15.9 to 315 mU/ml in ES HGSOC, 14.5 to 338 mU/ml in LS HGSOC, and 9.0 to 62.9 mU/ml in HC (Supplemental Fig. S11*B*). Overall, MUC1 exoproteins were significantly elevated in both ES HGSOC and LS HGSOC patients compared with HC (*p* < 0.05, two-tailed unpaired *t* test), further validating our proteomics data (Fig. 7*D*). AUC analysis shows discriminatory characteristics of MUC1 exoproteins with an AUC value of 0.840 for ES HGSOC *versus* HC and 0.860 for LS HGSOC *versus* HC, reflecting similar AUC values observed from the proteomics study (Fig. 7, *E* and *F*). We also evaluated the six candidate exoproteins in plasma-derived sEVs using ELISA but were unable to obtain reliable signals above background (data not shown), suggesting these proteins are present in low abundance in circulation. Detection of these exoproteins will likely require alternative, highly sensitive assay platforms, as we have recently described (37).

## Discussion

EVs offer distinct advantages as liquid biopsy biomarkers: (1) actively and continuously released, (2) multianalyte cargo, (3) cargo protected by lipid bilayer, (4) cargo reflective of parental cells, and (5) abundance in circulation. These features overcome the limitations of the contemporary biomarker candidates for liquid biopsy biomarkers: (1) circulating tumor cells, which are relatively low in abundance and reflect a later stage of tumor and (2) cell-free DNA, which is prone to degradation and can be contaminated by DNA released from apoptotic cells (8, 41). Hence, EVs are considered a new and promising candidate for liquid biopsy biomarkers (8, 9).

A critical step in EV-based biomarker research is isolating these nanoparticles from complex biofluids. Various EV isolation methods exploit size, density, surface property, charge, and dipole moment (9, 35, 42). Each of these methods can enrich different subpopulations of sEVs with varying levels of soluble protein impurities. Often, there is a trade-off between yield and purity (43, 44). Hence, the sEV isolation method should be carefully optimized based on the source of sEVs and downstream applications. Here, we used SEC to enrich for plasma sEVs, optimizing elution fractions that have minimal soluble impurities. Elution fractions were collected in an automated manner with an IZON AFC to ensure reproducibility across 70 plasma samples. sEV yields (total EV protein and particle count) did not differ across three independent cohorts but varied widely across patients in all three cohorts. This finding suggests that interpatient variability is the dominant determinant of total circulating sEV number and protein levels, and that these metrics are unlikely to provide discriminatory value in case *versus* control studies.

All cells continuously secrete EVs, and their cargo may carry the earliest biomarkers of disease. This feature can be highly relevant for silent cancers like HGSOC, where the survival beyond 10 years is only ∼18% in advanced disease (https://seer.cancer.gov/statfacts/html/ovary.html, Accessed on August 1, 2025). To date, no Food and Drug Administration–approved clinical tests can reliably detect the early onset of HGSOC. Previously, we have identified FT lineage–specific exoprotein biomarkers by profiling EVs from FT and HGSOC cells and tissue explants (22). In this current study, we extended this work using discovery proteomics in a case–control plasma cohort, identifying upregulated exoproteins in HGSOC that are associated with STIC precursor lesion and FT tissue lineage. Our two-pronged approach was (1) developing a disease-specific exoprotein spectral library (FT-HGSOC EV DIA/DDA library) and (2) crossreferencing the plasma exoproteome with available proteomic datasets to identify FT lineage and STIC-associated exoproteins upregulated in circulation. This enabled the detection of rare, disease-relevant exoproteins that might otherwise be below the detection of current technologies. Using this approach, we identified 26 exoproteins upregulated in ES HGSOC and 21 exoproteins upregulated in LS HGSOC that were exclusive to the disease-specific library (Fig. 3, *B* and *C*). Furthermore, comparison of the upregulated exoproteins in this study with previously published studies revealed candidate exoproteins of potential FT lineage and STIC association (Fig. 4). These candidates represent a valuable resource for future validation studies aimed at developing exoprotein-based diagnostic biomarkers for ES HGSOC originating from the FT.

Among the candidate exoprotein diagnostic biomarkers identified, we selected seven exoproteins (MYL6, GSTP1, PRDX6, MUC1, TTYH3, MYH14, and PTGS1) based on their potential to detect disease at its site of origin. We performed *in silico* analyses using available datasets (The Cancer Genome Atlas, GTEx, and Human Protein Atlas), alongside experimental validation to confirm their association with early cancer lesions, STIC, and FT lineage. MUC1, a membrane protein of the mucin family with O-glycosylation, is essential for forming protective mucous barriers on epithelial surfaces (45). It is involved in cell adhesion and cell signaling and is overexpressed in most adenocarcinomas, including HGSOC (46). TTYH3 is a membrane protein that plays a role in ion transport. In epithelial carcinoma, upregulation of TTYH3 has been shown to promote epithelial to mesenchymal transition through Wnt/β-catenin signaling, inhibit apoptosis, and promote cancer development and metastasis (47). In ovarian cancer, elevated expression of TTYH3 is linked to poor clinical outcomes (48). PRDX6 and GSTP1 are antioxidants involved in redox homeostasis. PRDX6 prevents NNMT ubiquitination and degradation as a nonenzymatic mechanism to promote ovarian cancer progression (49). PRDX6 has also been shown as a prognostic indicator in the effect of chemotherapy in ovarian cancer patients (50). GSTP1 detoxifies potentially genotoxic complexes, including chemotherapeutic drugs, and has been associated with chemoresistance against platinum-based chemotherapy in ovarian cancers (51, 52). PTGS1 plays a crucial role in the inflammatory response by converting arachidonic acid to prostaglandins (53). In HGSOC, PTGS1 shows selective overexpression compared with other subtypes of ovarian cancer (endometrioid, mucinous, and clear cell tumors) (54). In addition, pan-cancer analysis based on RNA-Seq data shows overexpression of the PTGS1 gene expression profile in cancer *versus* normal tissue, specifically in ovarian cancer compared with 21 other cancer types (Supplemental Fig. S12). These observations suggest that exoprotein PTGS1 can be explored as an HGSOC-specific diagnostic biomarker. MYL6 and MYH14 are myosins that are involved in cytoskeletal dynamics, motility, and contractility. In cancers, myosin is altered *via* phosphorylation, which supports proliferation, cell motility, and epithelial–mesenchymal transition (55, 56). Overall, these seven candidate exoproteins play roles in various cellular functions, including cell adhesion, cell signaling, ion transport, redox homeostasis, inflammatory response, cytoskeletal dynamics, and cell motility, which can contribute to malignancies relevant to cancer initiation and progression.

FT cell lines are a good model to study the early initiation of HGSOC. Immortalized FT secretory epithelial cell lines: FT240, FT194, and FT282 are commonly used in ovarian cancer research, specifically for studying the earliest stage of malignancy leading to ovarian cancer (7, 57, 58). FT282 cells are immortalized using vectors with human telomerase reverse transcriptase (hTERT) and genetically modified to express mutant TP53^R175H^ (59). FT194 cells are immortalized by hTERT and SV40 large T-antigen transduction, which disrupts the function of p53 and pRb (57, 58). FT240 cells are immortalized by hTERT, p53 shRNA, and CDK4^R24C^ (60). Immortalization of these cell lines was performed by hTERT expression to maintain telomerase activity, silencing the p53 (shRNA knockdown or mutation) and the CDK/CYCLIN/RB pathways to prevent cell cycle arrest and avoid senescence and apoptosis (58). These characteristics mimic the ES of malignancy in FT epithelial cells, which can lead to a p53 signature and STIC lesion. Our seven candidate exoproteins were expressed in these immortalized FT cell line–derived sEVs, and the expression was further retained in HGSOC cell line–derived sEVs, suggesting that these exoproteins that are upregulated in ES HGSOC might reflect the earliest stage of disease progression (Fig. 6*D*). This was further supported by our combinatorial panel of four exoprotein markers, which in the discovery cohort showed excellent discriminatory characteristics to detect ES HGSOC with an AUC of 0.975.

We further validated MUC1 exoprotein upregulation in both ES and LS HGSOC plasma-derived sEVs compared with HC. Previous studies have suggested MUC1 is a potential HGSOC-specific EV biomarker (18, 61, 62), but to our knowledge, this is the first study to validate its upregulation in plasma sEVs and confirm expression in tissues across disease stages (FT, HGSOC, and STIC precursor lesion). These findings support MUC1 exoprotein as a potential diagnostic biomarker for interrogating earlier stages of disease progression in HGSOC and MUC1 as a promising target for immunocapture-based enrichment of HGSOC-relevant sEVs from clinical samples. In addition to MUC1, our data identify six additional candidate exoproteins associated with early cancer lesions, FT lineage, and HGSOC.

Our analysis identified a cohort of exoproteins upregulated specifically in LS HGSOC cases but not in ES HGSOC cases (Fig. 4). While these exoproteins may have limited value as biomarkers for early detection, they could provide insights into mechanisms of disease progression and metastasis, given the role of EVs in paracrine and endocrine signaling (13, 63, 64). Notably, MUC16 exoproteins were upregulated only in LS HGSOC cases and not in ES HGSOC cases. MUC16 and its cleaved form, CA-125, are clinically used to monitor ovarian cancer, but they lack specificity and sensitivity (65, 66). Our findings suggest that MUC16 in circulating EVs may not serve as a diagnostic biomarker for ES HGSOC, underscoring the need for a more sensitive diagnostic biomarker for early detection.

The limitation of this study includes the lack of validation for most candidate exoproteins in plasma and the lack of a dedicated validation cohort. Future validation studies should focus on asymptomatic cases, where blood is drawn prior to diagnosis, and cases with pathologically confirmed STIC lesions before progression to HGSOC. Such studies will be essential to comprehensively evaluate the potential of candidate biomarkers for early detection. Although we validated expression of candidate exoproteins in cell line–derived sEVs using capillary Western blot, attempts to validate these proteins in plasma-derived sEVs by ELISA were largely unsuccessful because of their low abundance. This underscores the need for more sensitive single-EV detection technologies, which remains an ongoing research focus in our group. The detection range for ELISA was around 0.25 to 100 ng/ml based on the analyte. Compared with this, several highly sensitive single-EV analysis platforms, such as partition-less digital immunoassays, immuno-PCR–based assays, and droplet detection technologies, have demonstrated limits of detection as low as ∼9 EV/μl or protein concentrations down to 0.4 pg/ml (37, 67, 68). These single-EV detection technologies, if can be performed in high-throughput settings and process a high volume of EVs, can be a promising platform in detecting rare disease-relevant sEV in circulation. Another limitation of this study is the potential contamination of lipoproteins (>70 nm) or similarly sized biomolecules (50–200 nm) that can coisolate with sEVs.

In summary, from 70 plasma samples, we identified 52 exoproteins upregulated in ES HGSOC *versus* HC and 59 in LS HGSOC *versus* HC. Among these, 40 were potential FT lineage (source of origin for most HGSOC), 19 were STIC associated (precursor for HGSOC), and 23 were membrane associated (can be used for capture and detection assays). Expression of seven candidate exoproteins (MYL6, GSTP1, PRDX6, MUC1, TTYH3, MYH14, and PTGS1) was validated in (1) normal FT, FT with STIC lesion, and HGSOC tissue and (2) FT and HGSOC-cell culture–derived EVs. We further developed a combinatorial panel of four exoprotein biomarkers for ES HGSOC and validated upregulation of MUC1 exoproteins in plasma-derived sEVs from ES and LS HGSOC compared with HC. MUC1 could be used as a potential target to enrich disease-relevant sEV from clinical samples, and a colocalized detection of MUC1 exoproteins with other candidate exoproteins identified in this study may further improve the sensitivity and specificity of ES HGSOC detection. Together, we have identified circulating candidate exoprotein-based diagnostic biomarkers potentially associated with early cancer lesions, enabling investigation of the earliest changes happening in the HGSOC while the disease remains localized to the FT—at potentially curable stages.

## Data Availability

The MS proteomics data have been deposited to the ProteomeXchange Consortium *via* the PRIDE partner repository with the dataset identifier PXD067228.

## Supplemental Data

This article contains supplemental data including supplemental figures and supplemental tables.

## Conflict of Interest

A. K. G. serves as a scientific advisory board member to Exokeryx and receives research funding from Predicine and VITRAC Therapeutics. All the other authors declare no competing interests.

## References

[1] Lengyel E. (2010). Ovarian cancer development and metastasis. Am. J. Pathol..

[2] Arora T., Mullangi S., Lekkala M.R. (2022). StatPearls.

[3] Zhang S., Dolgalev I., Zhang T., Ran H., Levine D.A., Neel B.G. (2019). Both fallopian tube and ovarian surface epithelium are cells-of-origin for high-grade serous ovarian carcinoma. Nat. Commun..

[4] Bergsten T.M., Burdette J.E., Dean M. (2020). Fallopian tube initiation of high grade serous ovarian cancer and ovarian metastasis: mechanisms and therapeutic implications. Cancer Lett..

[5] Labidi-Galy S.I., Papp E., Hallberg D., Niknafs N., Adleff V., Noe M. (2017). High grade serous ovarian carcinomas originate in the fallopian tube. Nat. Commun..

[6] Shih I.-M., Wang Y., Wang T.-L. (2021). The origin of ovarian cancer species and precancerous landscape. Am. J. Pathol..

[7] Perets R., Wyant G.A., Muto K.W., Bijron J.G., Poole B.B., Chin K.T. (2013). Transformation of the fallopian tube secretory epithelium leads to high-grade serous ovarian cancer in Brca;Tp53;Pten models. Cancer Cell.

[8] Rayamajhi S., Sipes J., Tetlow A.L., Saha S., Bansal A., Godwin A.K. (2024). Extracellular vesicles as liquid biopsy biomarkers across the cancer journey: from early detection to recurrence. Clin. Chem..

[9] Tran H.L., Zheng W., Issadore D.A., Im H., Cho Y.-K., Zhang Y. (2025). Extracellular vesicles for clinical diagnostics: from bulk measurements to single-vesicle analysis. ACS Nano.

[10] Barlin M., Erdmann-Gilmore P., Mudd J.L., Zhang Q., Seymour R.W., Guo Z. (2023). Proteins in tumor-derived plasma extracellular vesicles indicate tumor origin. Mol. Cell Proteomics.

[11] Lischnig A., Bergqvist M., Ochiya T., Lässer C. (2022). Quantitative proteomics identifies proteins enriched in large and small extracellular vesicles. Mol. Cell Proteomics.

[12] Qiao Z., Zhang Y., Ge M., Liu S., Jiang X., Shang Z. (2019). Cancer cell derived small extracellular vesicles contribute to recipient cell metastasis through promoting HGF/c-Met pathway ∗[S]. Mol. Cell Proteomics.

[13] Sipes J., Zha D., Rayamajhi S., Bantis L.E., Madan R., Mitra A. (2025). Defining the ovarian cancer precancerous landscape through modeling fallopian tube epithelium reprogramming driven by extracellular vesicles. Cancer Res. Commun..

[14] Atay S., Wilkey D.W., Milhem M., Merchant M., Godwin A.K. (2018). Insights into the proteome of gastrointestinal stromal tumors-derived exosomes reveals new potential diagnostic biomarkers. Mol. Cell Proteomics.

[15] Atay S., Banskota S., Crow J., Sethi G., Rink L., Godwin A.K. (2014). Oncogenic KIT-containing exosomes increase gastrointestinal stromal tumor cell invasion. Proc. Natl. Acad. Sci..

[16] Crow J., Samuel G., Godwin A.K. (2019). Beyond tumor mutational burden: potential and limitations in using exosomes to predict response to immunotherapy. Expert Rev. Mol. Diagn..

[17] Hinestrosa J.P., Kurzrock R., Lewis J.M., Schork N.J., Schroeder G., Kamat A.M. (2022). Early-stage multi-cancer detection using an extracellular vesicle protein-based blood test. Commun. Med..

[18] Yokoi A., Ukai M., Yasui T., Inokuma Y., Hyeon-Deuk K., Matsuzaki J. (2023). Identifying high-grade serous ovarian carcinoma–specific extracellular vesicles by polyketone-coated nanowires. Sci. Adv..

[19] Jo A., Green A., Medina J.E., Iyer S., Ohman A.W., McCarthy E.T. (2023). Inaugurating high-throughput profiling of extracellular vesicles for earlier ovarian cancer detection. Adv. Sci..

[20] Zhang P., Zhou X., He M., Shang Y., Tetlow A.L., Godwin A.K. (2019). Ultrasensitive detection of circulating exosomes with a 3D-nanopatterned microfluidic chip. Nat. Biomed. Eng..

[21] Zha D., Rayamajhi S., Sipes J., Russo A., Pathak H.B., Li K. (2023). Proteomic profiling of fallopian tube-derived extracellular vesicles using a microfluidic tissue-on-chip System. Bioengineering.

[22] Trinidad C.V., Pathak H.B., Cheng S., Tzeng S.-C., Madan R., Sardiu M.E. (2023). Lineage specific extracellular vesicle-associated protein biomarkers for the early detection of high grade serous ovarian cancer. Sci. Rep..

[23] Kwong G.A., Ghosh S., Gamboa L., Patriotis C., Srivastava S., Bhatia S.N. (2021). Synthetic biomarkers: a twenty-first century path to early cancer detection. Nat. Rev. Cancer.

[24] Auber M., Svenningsen P. (2022). An estimate of extracellular vesicle secretion rates of human blood cells. J. Extracellular Biol..

[25] Ferguson S., Yang K.S., Zelga P., Liss A.S., Carlson J.C.T., del Castillo C.F. (2022). Single-EV analysis (sEVA) of mutated proteins allows detection of stage 1 pancreatic cancer. Sci. Adv..

[26] Rayamajhi S., Gibbs B.K., Sipes J., Pathak H.B., Bossmann S.H., Godwin A.K. (2024). Tracking small extracellular vesicles using a minimally invasive PicoGreen labeling strategy. ACS Appl. Bio. Mater..

[27] Hughes C.S., Moggridge S., Müller T., Sorensen P.H., Morin G.B., Krijgsveld J. (2019). Single-pot, solid-phase-enhanced sample preparation for proteomics experiments. Nat. Protoc..

[28] Searle B.C., Pino L.K., Egertson J.D., Ting Y.S., Lawrence R.T., MacLean B.X. (2018). Chromatogram libraries improve peptide detection and quantification by data independent acquisition mass spectrometry. Nat. Commun..

[29] Graw S., Tang J., Zafar M.K., Byrd A.K., Bolden C., Peterson E.C. (2020). proteiNorm – a user-friendly tool for normalization and analysis of TMT and label-free protein quantification. ACS Omega.

[30] Ritchie M.E., Phipson B., Wu D., Hu Y., Law C.W., Shi W. (2015). Limma powers differential expression analyses for RNA-sequencing and microarray studies. Nucleic Acids Res..

[31] Perez-Riverol Y., Bandla C., Kundu D.J., Kamatchinathan S., Bai J., Hewapathirana S. (2025). The PRIDE database at 20 years: 2025 update. Nucleic Acids Res..

[32] Gu Z., Eils R., Schlesner M. (2016). Complex heatmaps reveal patterns and correlations in multidimensional genomic data. Bioinformatics.

[33] Ge S.X., Jung D., Yao R. (2020). ShinyGO: a graphical gene-set enrichment tool for animals and plants. Bioinformatics.

[34] Bartha Á., Győrffy B. (2021). TNMplot.com: a web tool for the comparison of gene expression in normal, tumor and metastatic tissues. Int. J. Mol. Sci..

[35] Welsh J.A., Goberdhan D.C.I., O’Driscoll L., Buzas E.I., Blenkiron C., Bussolati B. (2024). Minimal information for studies of extracellular vesicles (MISEV2023): from basic to advanced approaches. J. Extracell. Vesicles.

[36] Xie Q., Zhou J., He C., Xu Y., Tao F., Hu M. (2024). Unlocking the intricacies: exploring the complex interplay between platelets and ovarian cancer. Crit. Rev. Oncol. Hematol..

[37] Wen Y., Li Y., Cheng S., Crow J., Samuel G., Vishwakarma V. (2025). Partition-less digital immunoassay using configurable topographic nanoarrays for extracellular vesicle diagnosis of ewing sarcoma. ACS Nano..

[38] Zhang P., Crow J., Lella D., Zhou X., Samuel G., Godwin A.K. (2018). Ultrasensitive quantification of tumor mRNAs in extracellular vesicles with an integrated microfluidic digital analysis chip. Lab. Chip.

[39] Wisztorski M., Aboulouard S., Roussel L., Duhamel M., Saudemont P., Cardon T. (2023). Fallopian tube lesions as potential precursors of early ovarian cancer: a comprehensive proteomic analysis. Cell Death Dis..

[40] Doberstein K., Spivak R., Reavis H.D., Hooda J., Feng Y., Kroeger P.T. (2022). L1CAM is required for early dissemination of fallopian tube carcinoma precursors to the ovary. Commun. Biol..

[41] Ma L., Guo H., Zhao Y., Liu Z., Wang C., Bu J. (2024). Liquid biopsy in cancer: current status, challenges and future prospects. Sig. Transduct. Target Ther..

[42] Suresh P.S., Zhang Q. (2025). Comprehensive comparison of methods for isolation of extracellular vesicles from human plasma. J. Proteome Res..

[43] Brennan K., Martin K., FitzGerald S.P., O’Sullivan J., Wu Y., Blanco A. (2020). A comparison of methods for the isolation and separation of extracellular vesicles from protein and lipid particles in human serum. Sci. Rep..

[44] Liangsupree T., Multia E., Riekkola M.-L. (2021). Modern isolation and separation techniques for extracellular vesicles. J. Chromatogr. A.

[45] Chen W., Zhang Z., Zhang S., Zhu P., Ko J.K.-S., Yung K.K.-L. (2021). MUC1: structure, function, and clinic application in epithelial cancers. Int. J. Mol. Sci..

[46] Deng J., Wang L., Chen H., Li L., Ma Y., Ni J. (2013). The role of tumour-associated MUC1 in epithelial ovarian cancer metastasis and progression. Cancer Metastasis Rev..

[47] Xue W., Dong B., Zhao Y., Wang Y., Yang C., Xie Y. (2021). Upregulation of TTYH3 promotes epithelial-to-mesenchymal transition through Wnt/β-catenin signaling and inhibits apoptosis in cholangiocarcinoma. Cell Oncol..

[48] Zhou J., Xu Y., Chen X., Chen F., Zhang J., Zhu X. (2021). Elevated expression of tweety homologue 3 predicts poor clinical outcomes in ovarian cancer. J. Cancer.

[49] Wu X., Luo L., Wang M., Dong L., Fan J., Zeng Y. (2025). PRDX6 prevents NNMT ubiquitination and degradation as a nonenzymatic mechanism to promote ovarian cancer progression. Adv. Sci..

[50] Li S., Hu X., Ye M., Zhu X. (2018). The prognostic values of the peroxiredoxins family in ovarian cancer. Biosci. Rep..

[51] Cui J., Li G., Yin J., Li L., Tan Y., Wei H. (2020). GSTP1 and cancer: expression, methylation, polymorphisms and signaling (Review). Int. J. Oncol..

[52] Sawers L., Ferguson M.J., Ihrig B.R., Young H.C., Chakravarty P., Wolf C.R. (2014). Glutathione S-transferase P1 (GSTP1) directly influences platinum drug chemosensitivity in ovarian tumour cell lines. Br. J. Cancer.

[53] Cebola I., Peinado M.A. (2012). Epigenetic deregulation of the COX pathway in cancer. Prog. Lipid Res..

[54] Wilson A.J., Fadare O., Beeghly-Fadiel A., Son D.-S., Liu Q., Zhao S. (2015). Aberrant over-expression of COX-1 intersects multiple pro-tumorigenic pathways in high-grade serous ovarian cancer. Oncotarget.

[55] Kozole S.L., Beningo K.A. (2024). Myosin light chains in the progression of cancer. Cells.

[56] Ouderkirk J.L., Krendel M. (2014). Non-muscle myosins in tumor progression, cancer cell invasion, and metastasis. Cytoskeleton.

[57] Karst A.M., Levanon K., Drapkin R. (2011). Modeling high-grade serous ovarian carcinogenesis from the fallopian tube. Proc. Natl. Acad. Sci..

[58] Mei J., Tian H., Huang H.-S., Hsu C.-F., Liou Y., Wu N. (2021). Cellular models of development of ovarian high-grade serous carcinoma: a review of cell of origin and mechanisms of carcinogenesis. Cell Prolif..

[59] Karst A.M., Jones P.M., Vena N., Ligon A.H., Liu J.F., Hirsch M.S. (2014). Cyclin E1 deregulation occurs early in secretory cell transformation to promote formation of fallopian tube–derived high-grade serous ovarian cancers. Cancer Res..

[60] Karst A.M., Drapkin R. (2012). Primary culture and immortalization of human fallopian tube secretory epithelial cells. Nat. Protoc..

[61] Salem D.P., Bortolin L.T., Gusenleitner D., Grosha J., Zabroski I.O., Biette K.M. (2024). Colocalization of cancer-associated biomarkers on single extracellular vesicles for early detection of cancer. J. Mol. Diagn..

[62] Winn-Deen E.S., Bortolin L.T., Gusenleitner D., Biette K.M., Copeland K., Gentry-Maharaj A. (2024). Improving specificity for ovarian cancer screening using a novel extracellular vesicle–based blood test: performance in a training and verification cohort. J. Mol. Diagn..

[63] Hoshino A., Costa-Silva B., Shen T.-L., Rodrigues G., Hashimoto A., Tesic Mark M. (2015). Tumour exosome integrins determine organotropic metastasis. Nature.

[64] Zhao L., Ma X., Yu J. (2021). Exosomes and organ-specific metastasis. Mol. Ther. Methods Clin. Dev..

[65] Topalak O., Saygili U., Soyturk M., Karaca N., Batur Y., Uslu T. (2002). Serum, pleural effusion, and ascites CA-125 levels in ovarian cancer and nonovarian benign and malignant diseases: a comparative study. Gynecol. Oncol..

[66] Sevinc A., Adli M., Kalender M.E., Camci C. (2007). Benign causes of increased serum CA-125 concentration. Lancet Oncol..

[67] Shami-Shah A., Norman M., Walt D.R. (2023). Ultrasensitive protein detection technologies for extracellular vesicle measurements. Mol. Cell Proteomics.

[68] Reynolds D.E., Pan M., Yang J., Galanis G., Roh Y.H., Morales R.-T.T. (2023). Double digital assay for single extracellular vesicle and single molecule detection. Adv. Sci..

