# Supplementary material for: Circulating Extracellular Vesicle Protein Biomarkers for the Early Detection of High-Grade Serous Ovarian Cancer
Source: Mol Cell Proteomics. 2026 Jan 8;25(2):101508. doi: 10.1016/j.mcpro.2026.101508 (PMC12891884; doi:10.1016/j.mcpro.2026.101508)
Supplement: Supplemental Data [file mmc1.docx]

**Supplemental Data for:**

**Circulating extracellular vesicle protein biomarkers for the early detection of high-grade serous ovarian cancer**

Sagar Rayamajhi^1^, Jared Sipes^1,2^, Bidii Ngala^1^, Amrita Mitra^1^, Meizhang Li^1^, Camille V. Trinidad^1,3^, Wei Cui^1^, Mohammod Mahmudur Rahman^4^, Foyez Ahmmed^4^, Leonidas E. Bantis^4^, Mihaela E. Sardiu^4^, Dennis W. Province^5^, Harsh B. Pathak^1,6^, Andrew K. Godwin^1,2,6,7^

^1^Department of Pathology and Laboratory Medicine, University of Kansas Medical Center, Kansas City, KS 66160, USA

^2^Bioengineering Program, The University of Kansas, Lawrence, KS, 64111, USA

^3^Department of Biomedical Engineering, Vanderbilt University, Nashville, TN, 37212, USA

^4^Department of Biostatistics and Data Science, University of Kansas Medical Center, Kansas City, KS 66160, USA

^5^Department of Biochemistry and Microbiology, University of Arkansas for Medical Science, Little Rock, AR 72205, USA

^6^Kansas Institute for Precision Medicine, University of Kansas Medical Center, Kansas City, KS 66160, USA

^7^University of Kansas Cancer Center, University of Kansas Medical Center, Kansas City, KS 66160, USA

Correspondence:

Andrew K. Godwin, PhD

Department of Pathology & Laboratory Medicine

3901 Rainbow Blvd

University of Kansas Medical Center

Kansas City, KS 66160

(913) 945-8568

[agodwin@kumc.edu](mailto:agodwin@kumc.edu)

**Supplemental Table S1. Clinical information for the 70 patients included in the cases and controls and 9 patients included in the IHC tissue experiment.**

**Supplemental Table S2. Antibody information used for validation of candidate exo-proteins using (A) IHC and (B) capillary-based western blot (Jess).**

**Supplemental Table S3. Normalized intensity of exo-proteins identified across 70 samples, reported for each library in a separate worksheet tab in excel file.**

**Supplemental Table S4.** **Quantitative peptide reports of exo-proteins identified across 70 samples, reported for each library in a separate worksheet tab in excel file.**

**Supplemental Table S5. MQ evidence file for Plasma EV DDA library.**

**Supplemental Table S6. MQ evidence file for FT-HGSOC EV DDA library.**

**Supplemental Table S7. Quantitative peptide reports for Plasma EV DIA library.**

**Supplemental Table S8. Quantitative peptide reports for FT-HGSOC EV DIA library.**

**Supplemental Table S9. Gene IDs of (A) 52 upregulated exo-proteins in ES HGSOC vs HC and (B) 59 upregulated exo-proteins in LS HGSOC vs HC.**

**Supplemental Table S10. Details of seven candidate exo-protein biomarkers.**


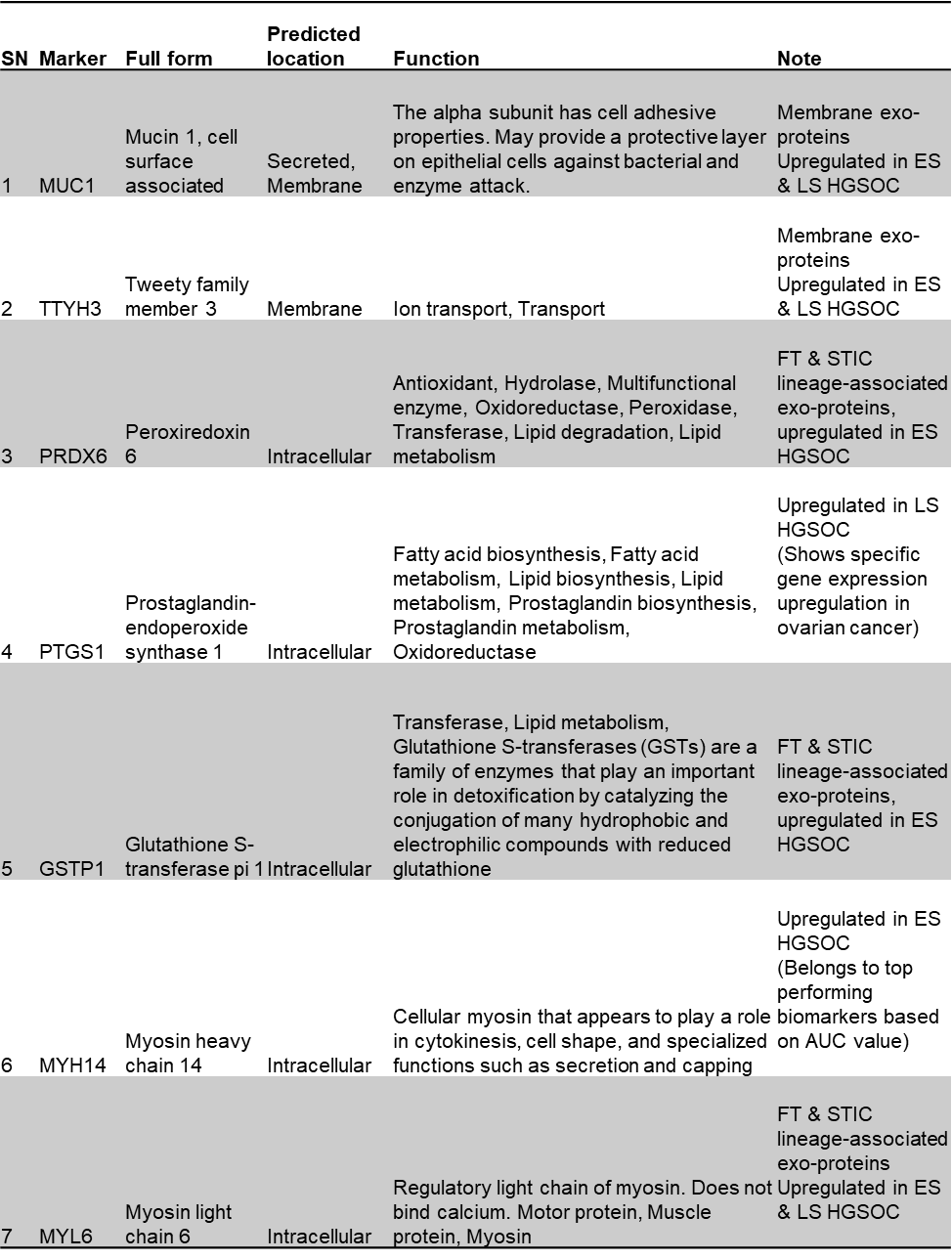


**Supplemental Table S11. AUC table of 7 candidate biomarker along with panel of combination biomarker in early-stage HGSOC vs HC cohort.**


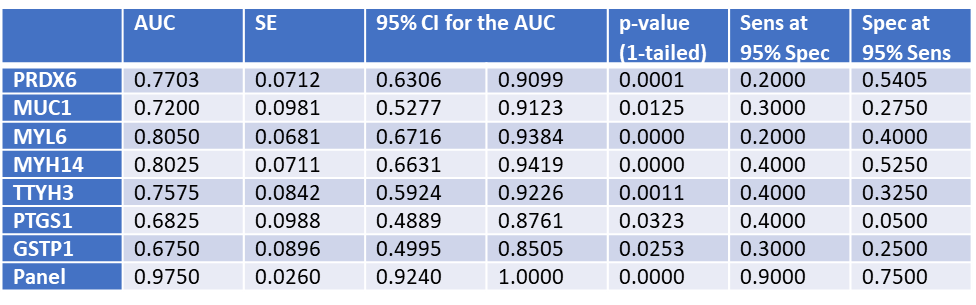


**Supplemental Table S12. The main biological pathway associations for the seven candidate markers, highlighting the four panel exo-proteins.**

**Supplemental Table S13. Biological pathway associations for seven candidate diagnostic biomarkers as determined by ConsensusPathDB analysis. For each marker, major pathways, representative example processes, and relevant ConsensusPathDB pathway identifiers are provided.**

**
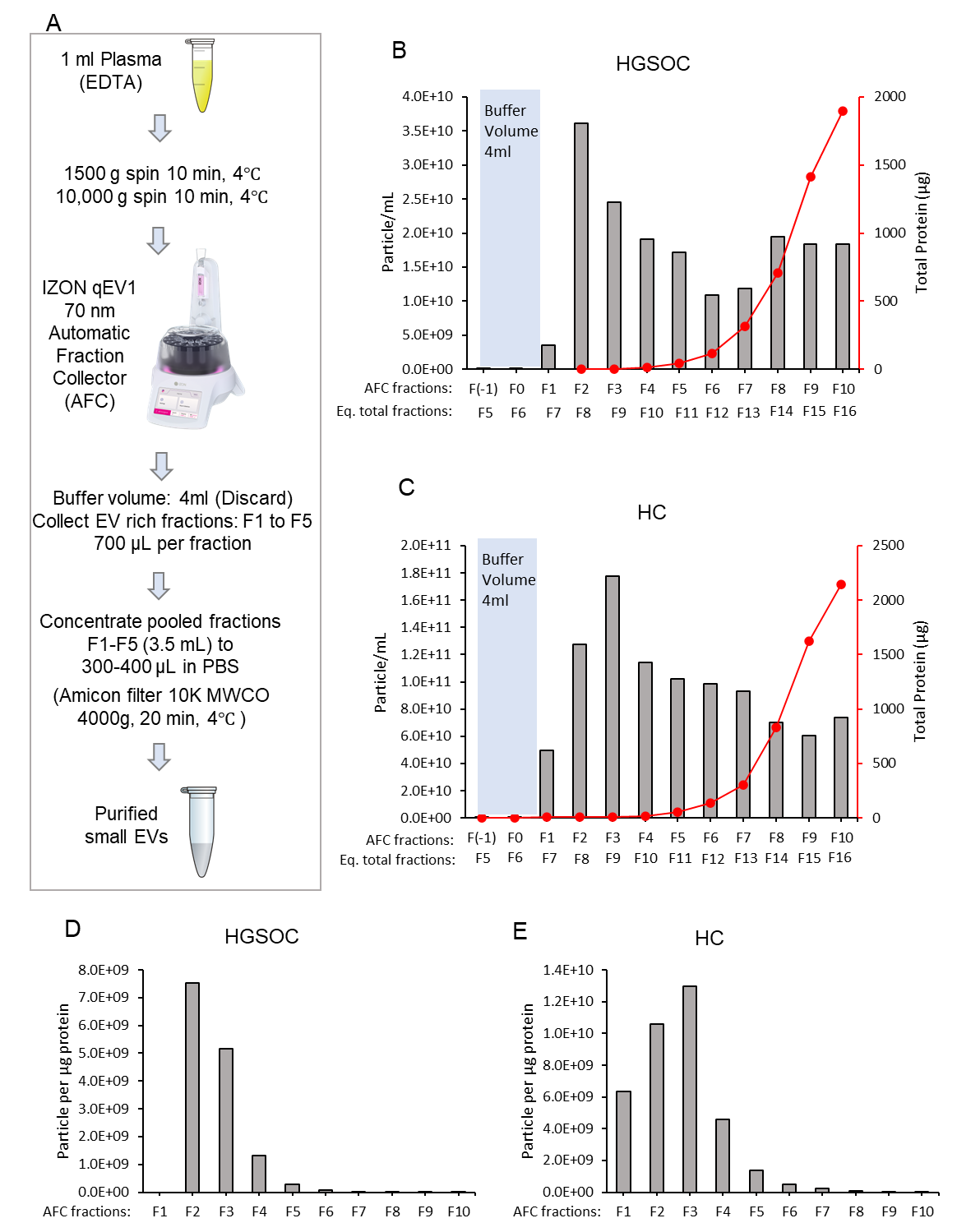
**

**Supplemental Figure S1**. **Optimization of SEC protocol for enrichment of sEVs. A**) Schematic showing small EV isolation protocol from plasma using size exclusion chromatography (SEC). SEC was run using the IZON qEV1 70 nm SEC column on the IZON automatic fraction collector (AFC), and elution fraction was optimized to enrich small EVs while avoiding soluble proteins. 4 ml was set as buffer volume, which is the initial elution from SEC that contains buffer only without EVs and is discarded. Elution fraction (700 µL per fraction) was collected following the 4 ml buffer volume. **B,C**) Characterization of 10 elution fractions (F1 to F10) for EV particle and protein content following a 1 ml plasma SEC run from HGSOC and HC samples, respectively. Both plasma samples show a similar trend in EV enrichment. EVs are detected in F1, and peak concentration is reached in F2-F3, while total protein starts to spike significantly after F6 and beyond, suggesting elution of soluble protein impurities from F6. Based on the data, F1 to F5 were selected as small EV-enriched fractions. A total fraction equivalent is also shown in the figure, which represents the fraction number including the buffer volume. F1 to F5 in AFC is equivalent to F7 to F11 in total fraction. **D,E**) AFC elution fractions (F1-F10) were normalized based on EV protein content (particles per µg protein) and plotted to visualize EV enrichment in HGSOC and HC plasma samples, respectively. Peak EV enrichment was observed in F2-F3 with a range of F1-F5, further suggesting that AFC fractions F1-F5 are enriched in small EVs.


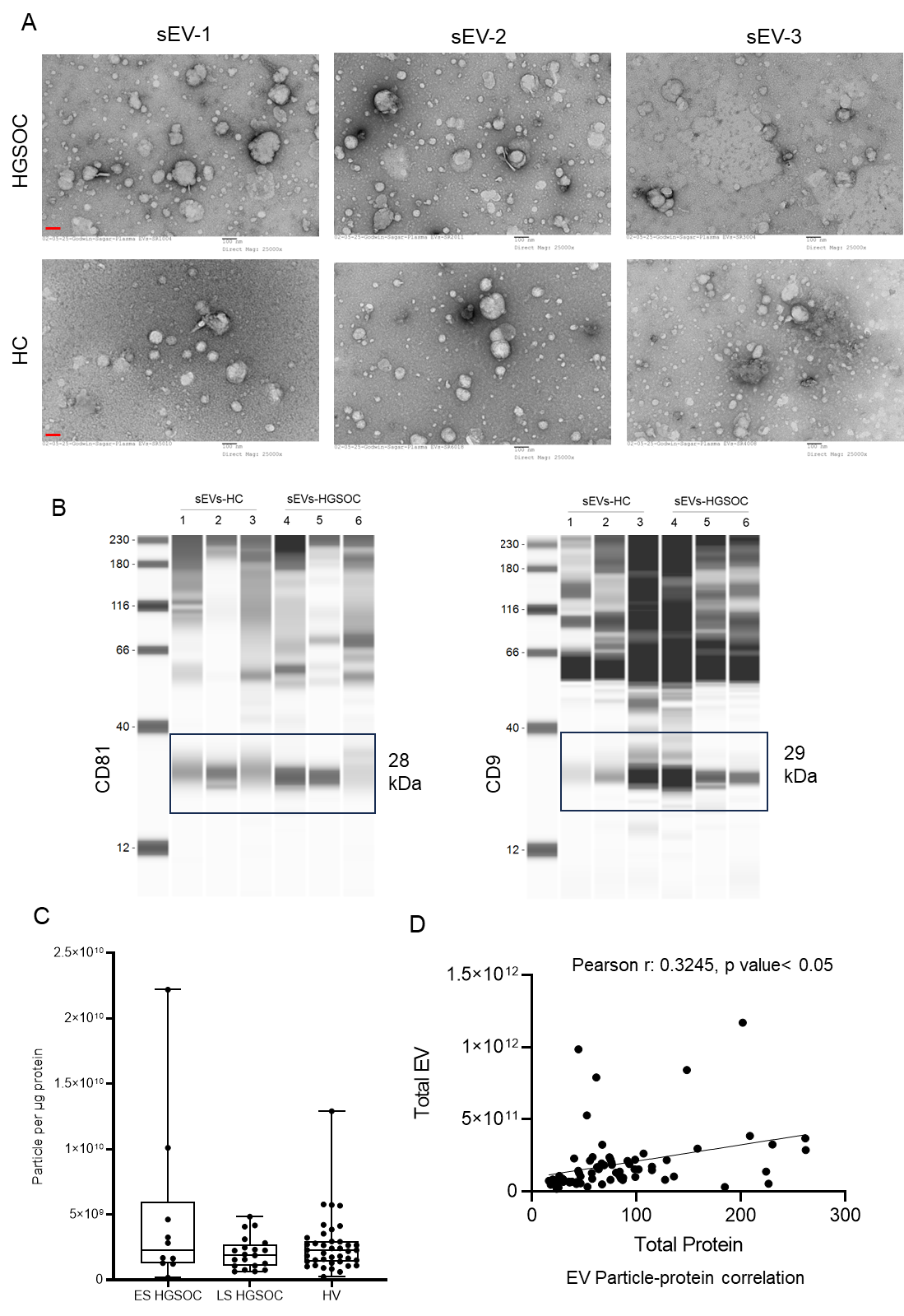


**Supplemental Figure S2**. **Characterization of plasma-derived sEVs.**  **A**) Transmission electron micrograph of representative plasma-derived sEV showing morphological characteristics (Magnification: 25000X, scale bar: 100 nm). Three representative sEV samples from HGSOC cases and three representative sEV samples from HC cases are shown. **B**) Full blot image of capillary-based western blot in six representative plasma-derived EVs for EV marker proteins CD81 and CD9, respectively. **C**) Quantification of EV normalized to the protein content (particles per µg protein) in case vs control cohort. **D**) Correlation of EV protein content with particle number in 70 plasma-derived sEV samples showing overall weak, but statistically significant, correlation (Pearson r: 0.3245, p value<0.05).


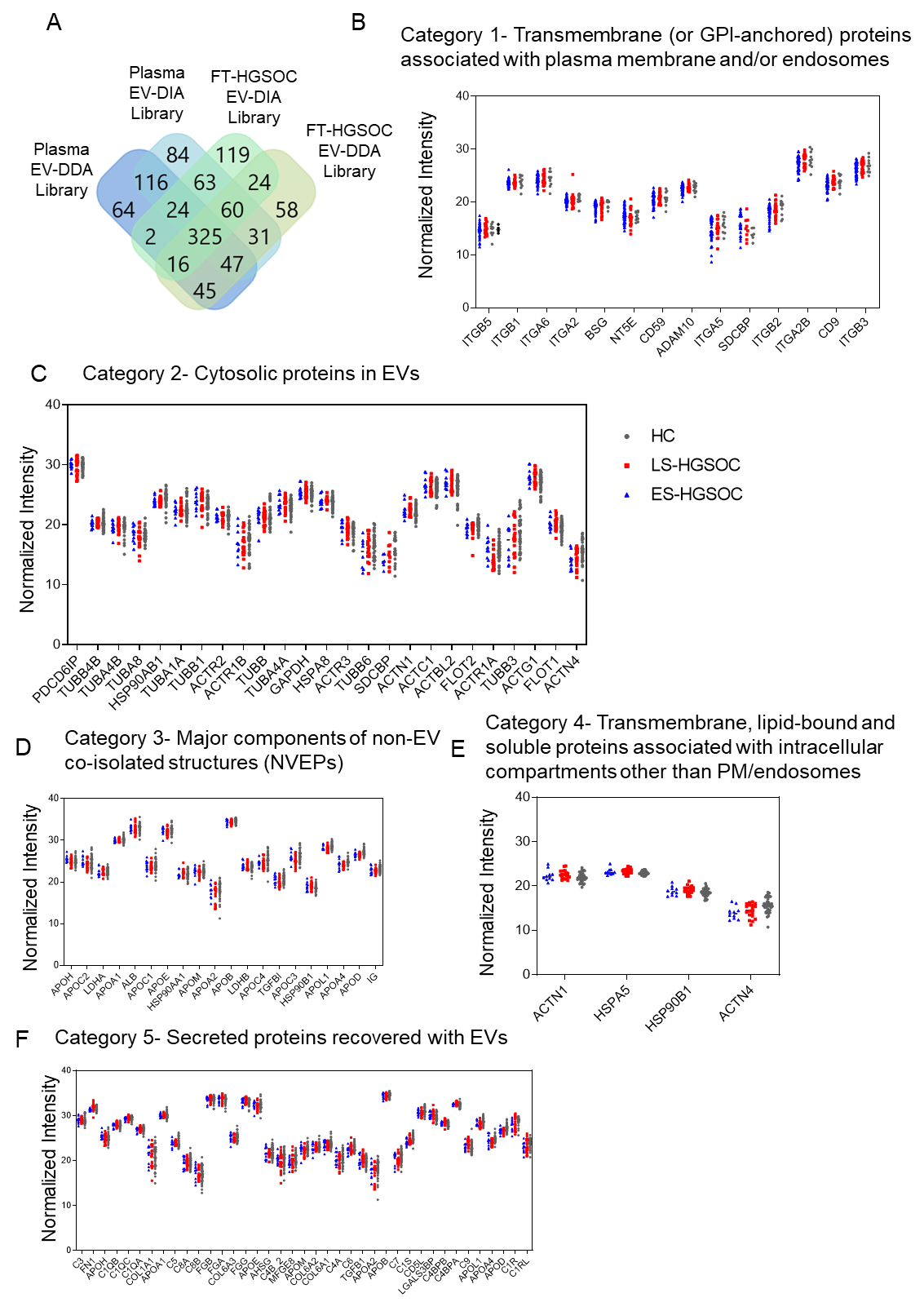


**Supplemental Figure S3. Identification of EV-associated protein categories in ES HGSOC, LS HGSOC, and HC cohorts. A**) Comparison of the total exo-proteins identified in each of the four spectral libraries across 70 plasma-derived sEV samples. **B-F**) Five major categories of EV-associated proteins identified in the plasma-derived sEV proteome by LC-MS/MS. The list of 5 categories of EV-associated protein is based upon guidelines by the International Society of Extracellular Vesicles (*Welsh et al. JEV 2023*).


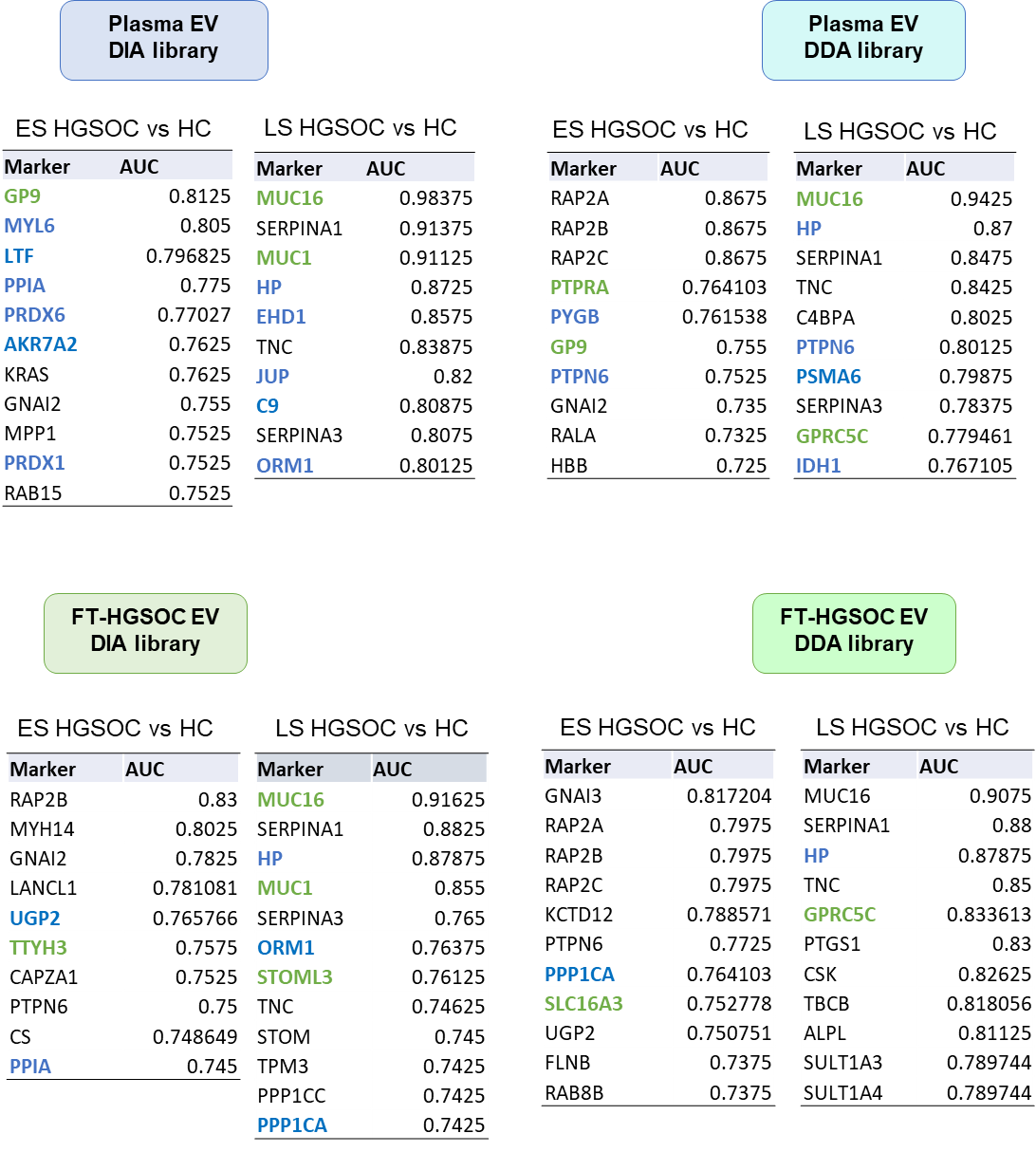


**Supplemental Figure S4**. **Top exo-protein biomarkers based on AUC value.** List of top exo-proteins biomarkers ranked by their discriminatory power between cases and controls, quantified by AUC. AUC values were calculated based on normalized intensity of exo-proteins identified by LC-MS/MS, highlighting top-performing exo-proteins in ES HGSOC vs HC and LS HGSOC vs HC across four different spectral libraries. Green text denotes membrane associated exo-proteins, and blue denotes potential FT lineage-associated exo-proteins.


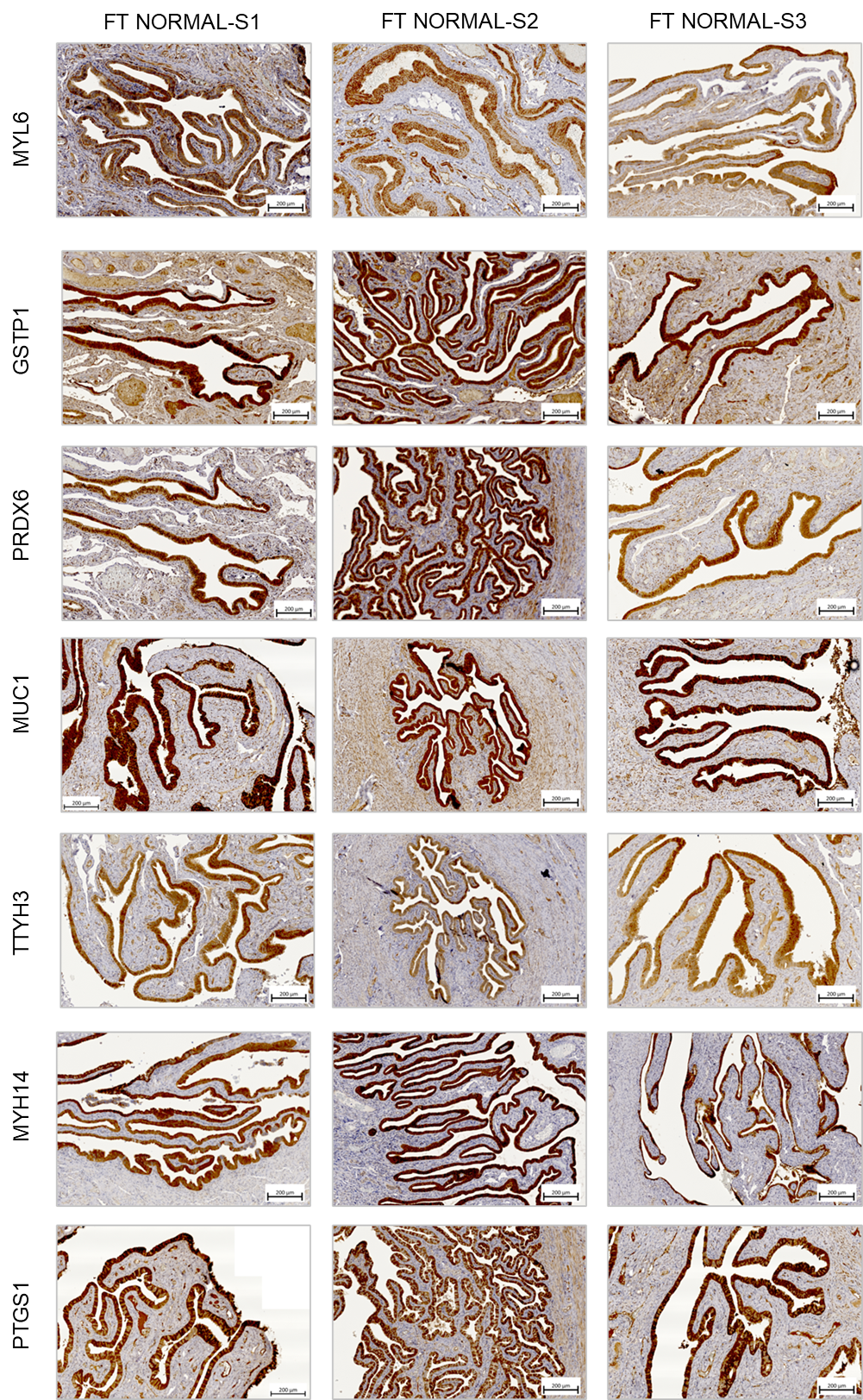


**Supplemental Figure S5. Expression patterns of the 7 prioritized exo-proteins in fallopian tube tissue.** IHC staining of the 7 exo-proteins in FFPE fallopian tube tissue sections. Representative images shown from three biological replicates each corresponding to a different patient sample. Scale bar: 200 µm.


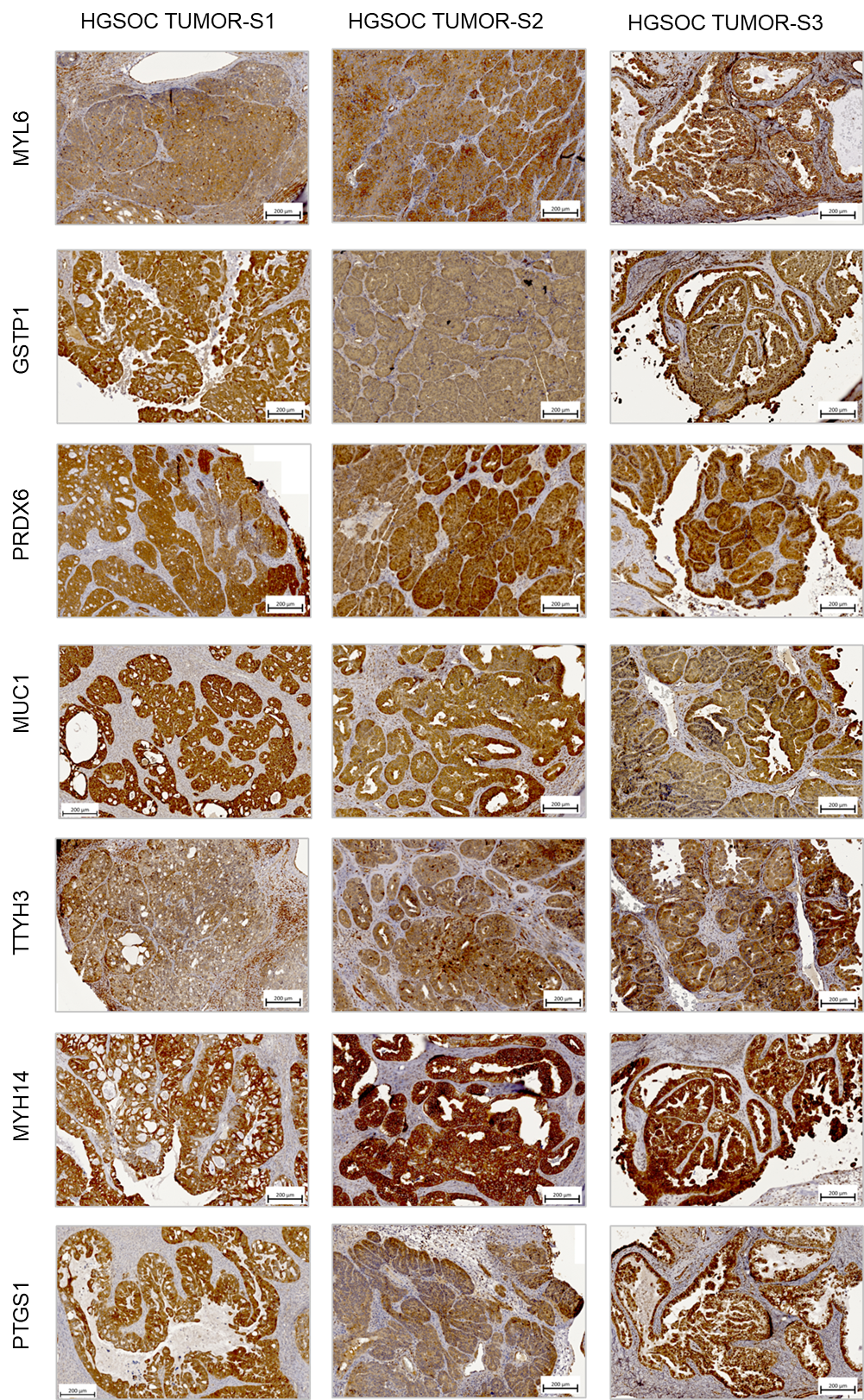


**Supplemental Figure S6. Expression patterns of the 7 prioritized exo-proteins in high-grade serous ovarian tissue.** IHC staining of the 7 exo-proteins in FFPE high-grade serous ovarian cancer tissue sections. Representative images shown from three biological replicates each corresponding to a different patient sample. Scale bar: 200 µm.


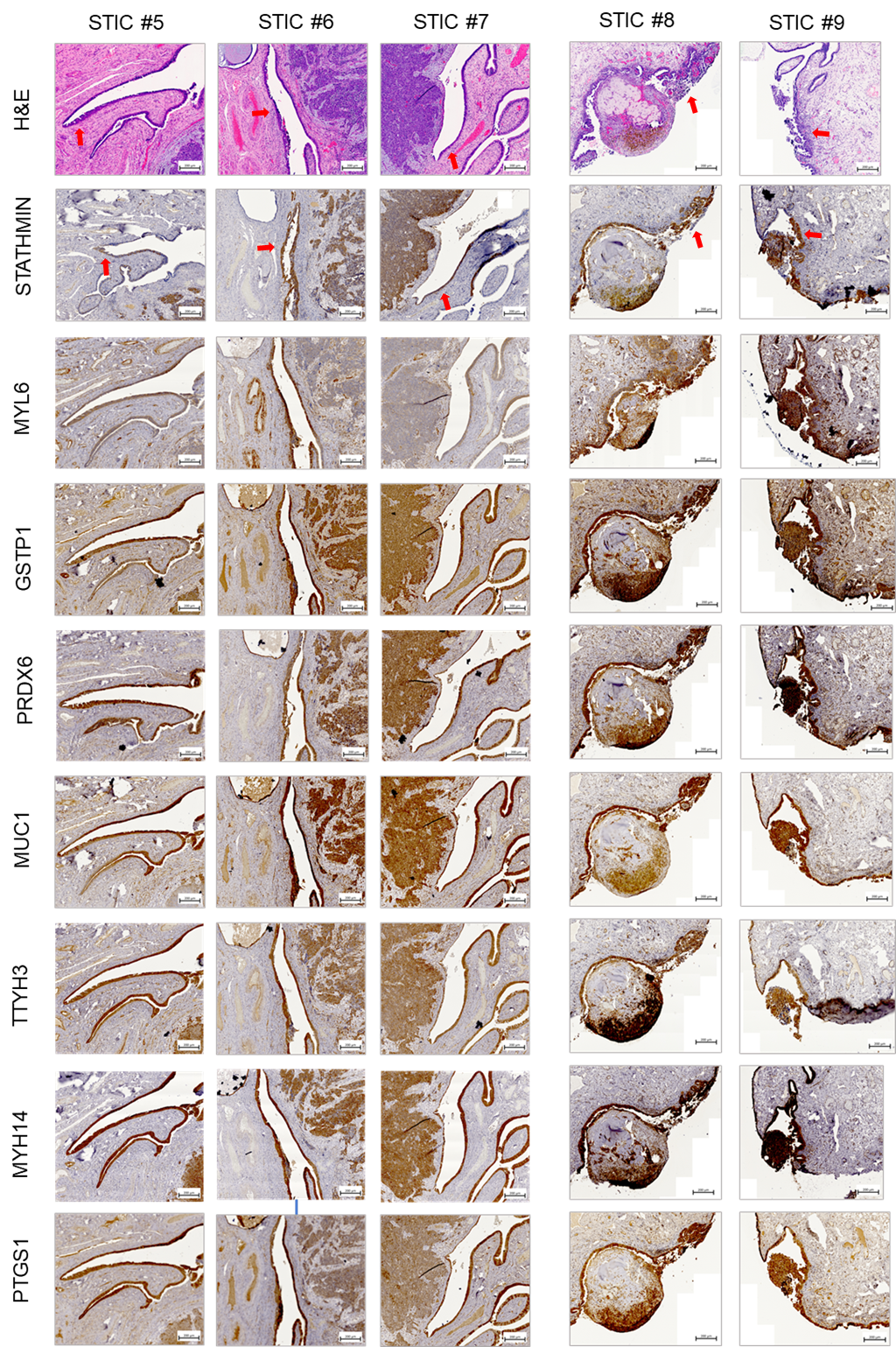


**Supplemental Figure S7**. **Expression patterns of the 7 prioritized exo-proteins in STIC lesions**. IHC staining of the 7 exo-proteins in FFPE of fallopian tube tissues with STIC lesions. Representative images shown are from 8 distinct STIC sites (red arrow) from 2 different patients. Scale bar: 200 µm.


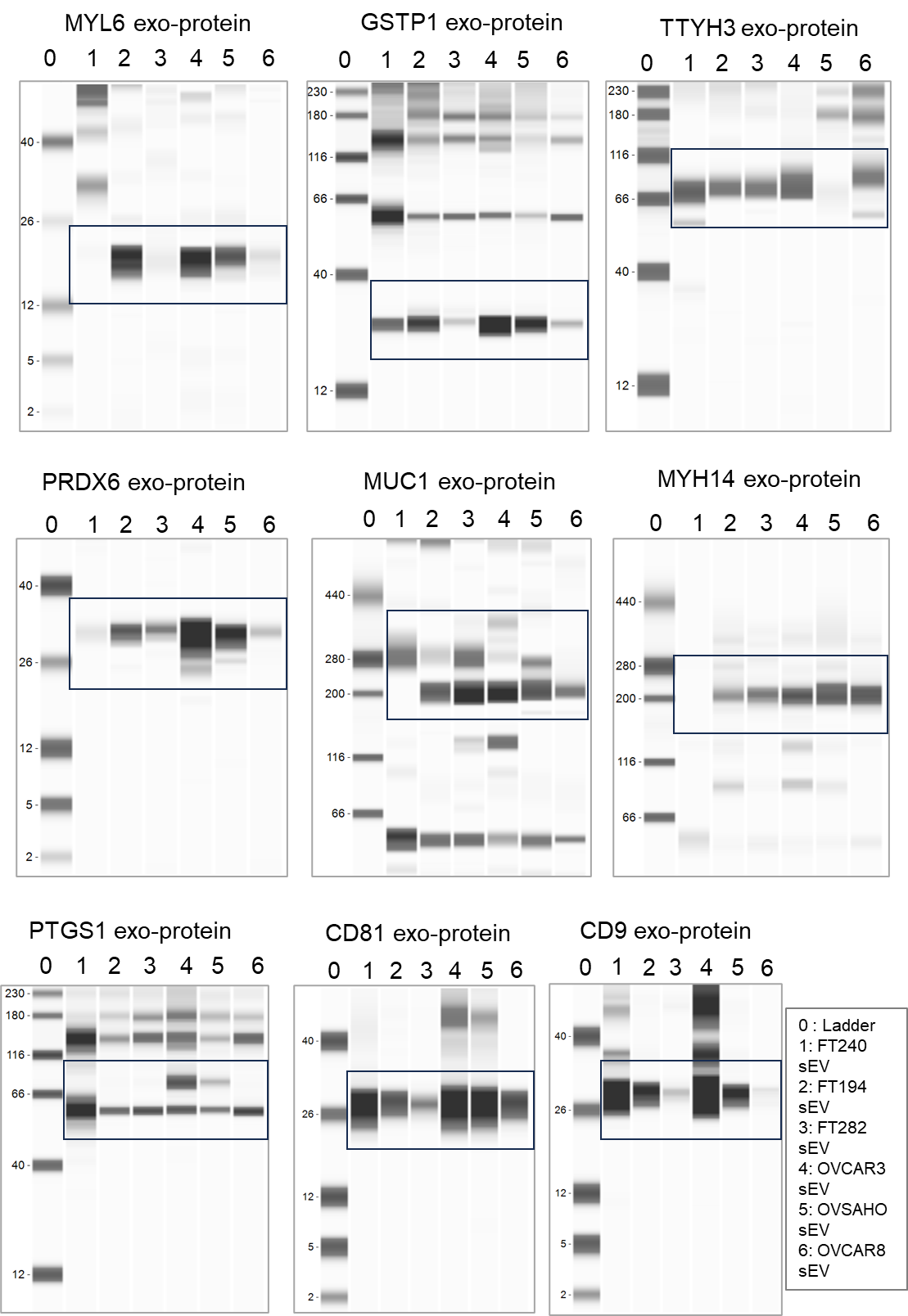


**Supplemental Figure S8**. **Full blot images of capillary-based western blot for the 7 candidate exo-proteins in cell-derived sEVs.** CD81 and CD9 are common EV-associated protein markers.


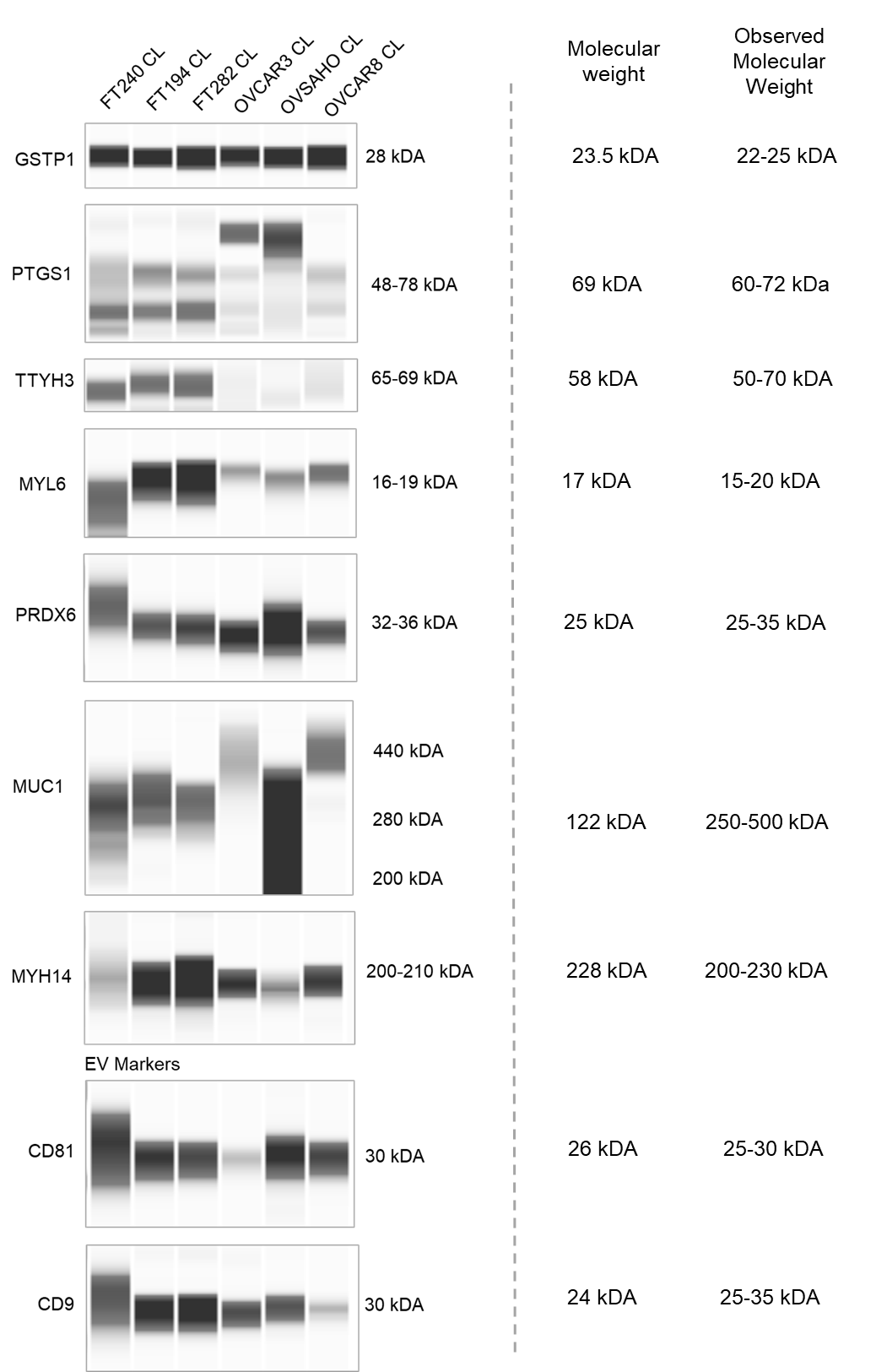


**Supplemental Figure S9**. **Expression of 7 candidate exo-proteins in fallopian tube and ovarian cancer cell lysates.** Expression of 7 candidate exo-proteins was validated in 3 different fallopian tube cell lines (FT240, FT194, FT282) and 3 different high-grade serous cell lines (OVCAR3, OVSAHO, OVCAR8) using cell lysates (600 µg/mL) and capillary-based western blot. CD81 and CD9 are common EV-associated protein markers.

**
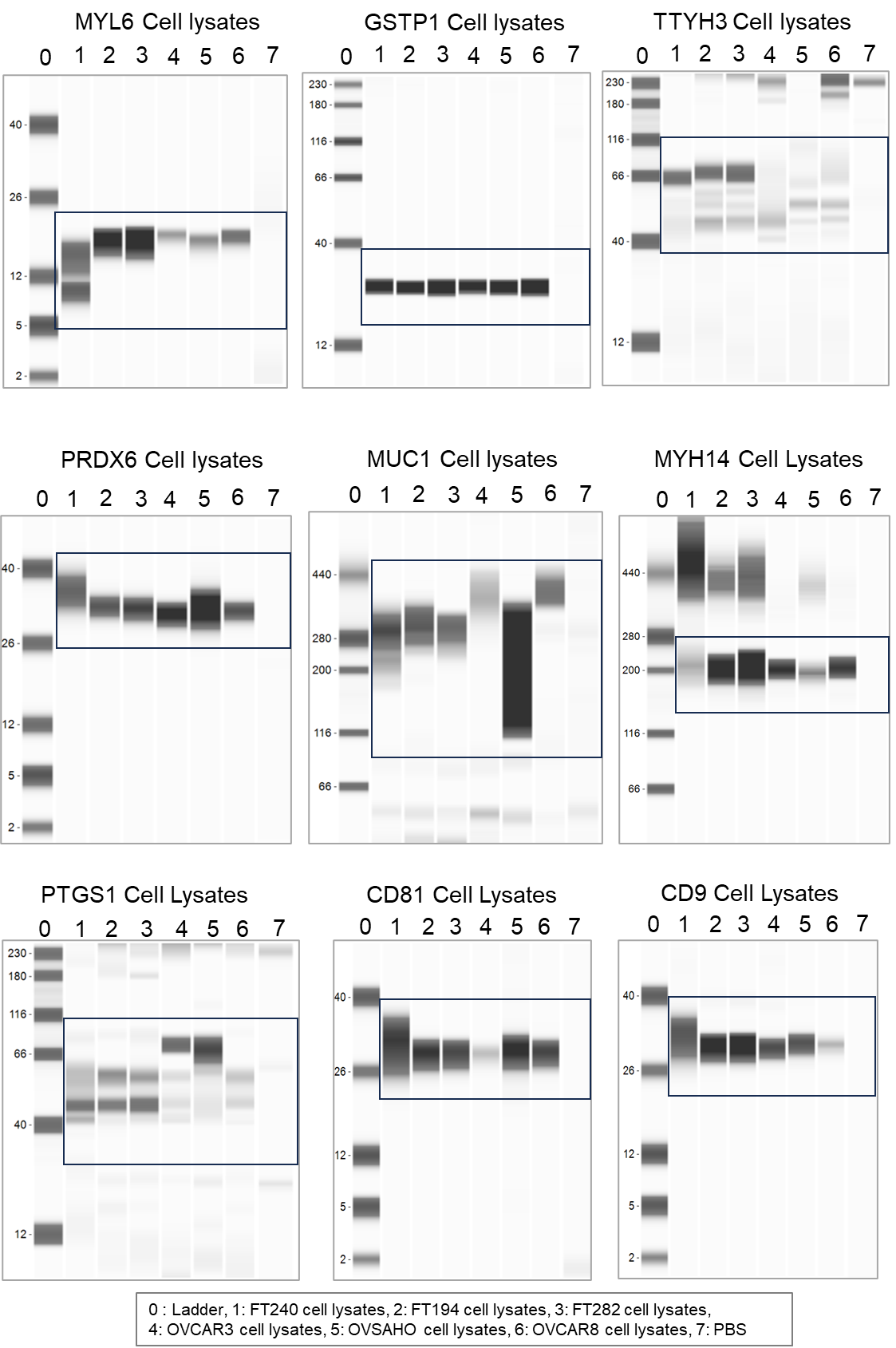
**

**Supplemental Figure S10**. **Full blot images of capillary-based western blot for the 7 candidate exo-proteins in cell lysates.** Cell lysates are used as positive control and PBS as negative control. CD81 and CD9 are common EV-associated protein markers.


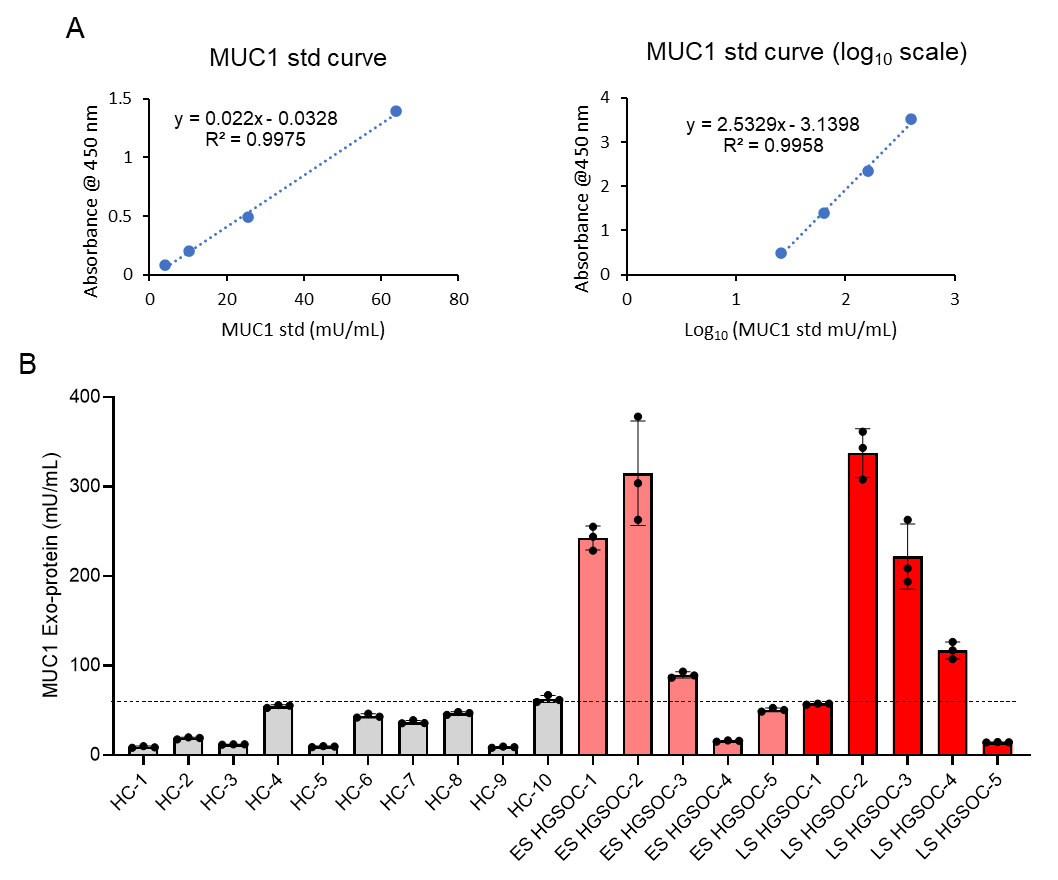


**Supplemental Figure S11**. **Assessment of MUC1 exo-proteins in plasma-derived sEVs**. A) Standard curve of MUC1 proteins using sandwich ELISA. Standard curves are plotted in linear scale MUC1 concentration (mU/mL) vs absorbance value at 450 nm (for low MUC1 concentration samples) and log10 scale MUC1 concentration (mU/mL) vs absorbance at 450 nm (for high MUC1 concentration samples). B) Quantification of MUC1 exo-proteins across 20 plasma-derived sEVs (10 healthy controls, 5 early-stage HGSOC, 5 late-stage HGSOC). MUC1 concentration (mU/mL) was quantified using blank-subtracted absorbance at 450 nm and compared with the MUC1 standard curve. Dotted line at ~63 mU/mL represents cut-off point to separate HGSOC from HC based on MUC1 concentration.


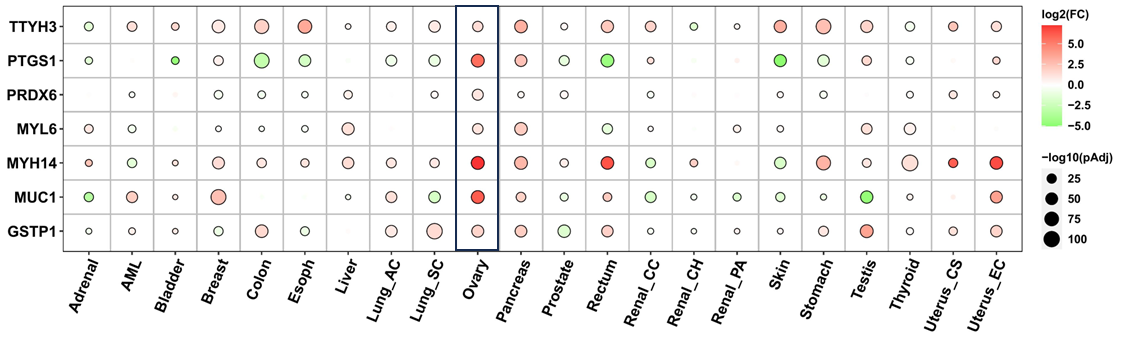


**Supplemental Figure S12**. **Pan-cancer expression profile of the genes encoding the seven prioritized candidate exo-protein biomarkers in various cancer types.** The pan-cancer heatmap analysis shows log2 FC values of tumor/normal RNA-Seq data. Red color represents higher expression in the tumor, while the blue color indicates higher expression in normal tissues. The sizes of the circles are inversely proportional to the adjusted P values. Figure is created from “TNMplot: differential gene expression analysis in Tumor, Normal, and Metastatic tissues (tnmplot.com)”.
